# scSorter: assigning cells to known cell types according to marker genes

**DOI:** 10.1186/s13059-021-02281-7

**Published:** 2021-02-22

**Authors:** Hongyu Guo, Jun Li

**Affiliations:** Department of Applied and Computational Mathematics and Statistics, University of Notre Dame, 102 Crowley Hall, Notre Dame, USA

**Keywords:** Cell type assignment, Single-cell RNA-seq, Marker genes, Clustering

## Abstract

On single-cell RNA-sequencing data, we consider the problem of assigning cells to known cell types, assuming that the identities of cell-type-specific marker genes are given but their exact expression levels are unavailable, that is, without using a reference dataset. Based on an observation that the expected over-expression of marker genes is often absent in a nonnegligible proportion of cells, we develop a method called scSorter. scSorter allows marker genes to express at a low level and borrows information from the expression of non-marker genes. On both simulated and real data, scSorter shows much higher power compared to existing methods.

**Supplementary Information:**

The online version contains supplementary material available at (10.1186/s13059-021-02281-7).

## Background

Single-cell RNA sequencing (scRNA-seq) quantifies gene expression of a large number of individual cells, which is then used to identify and describe different types of cells. These cell types include not only novel cell types but also known cell types, that is, cell types that have been previously discovered and studied. Assigning cells to known cell types is important as the proportional composition of known cell types helps to understand how heterogeneous tissues function. Assigning cells to known cell types can also facilitate the search of new cell types as the search can focus on “unknown” cells, i.e., cells that cannot be assigned to known cell types. Moreover, as large-scale efforts/projects such as Human Cell Atlas [1] proceed, most (non-rare) cell types will become “known,” and thus, the task of assigning cells to known cell types will become even more prevalent.

Cell-type assignment can be done manually: after clustering cells into cell types using computational clustering algorithms such as Leiden [2] or Louvain [3] on a nearest neighbor graph (both implemented in Seurat [4]) and SC3 [5], label the cell clusters to known cell types based on manual examination of differentially expressed genes specific to each cluster [6]. The subjectivity of manual examination is a problem, which can be eliminated by fully automated computational approaches.

Most of current computational approaches utilize a “reference” dataset, i.e., a previously studied dataset where these known cell types are present. They train a classifier using the reference dataset and apply this classifier to the dataset in hand. Many well-designed classifiers that carefully accommodate features and characteristics of single-cell data have been developed and used (e.g., [7–14]). The success of these approaches, however, depends on the availability of a reference dataset, which, ideally, should be of high quality and resembles the data in hand in the sense of the samples being prepared in a similar manner and the expression being generated using a similar technique, in order to minimize the influential systematic differences between datasets (e.g., batch effect), which is often a concern in scRNA-seq data (e.g., [15]).

Although the availability of a suitable reference dataset can be limited, knowledge about known cell types has been accumulated over the years, which is often in the form like “in cell type A, genes a, b, and c are highly expressed.” Such genes, i.e., genes that are over-expressed in a particular cell type, are often called “marker genes” of the cell type. Typically, the knowledge about marker genes is “qualitative” instead of “quantitative.” That is, they are known to be expressed at higher levels in the corresponding cell types but their exact expression levels are unknown. This is because these marker genes may have been discovered using various techniques, from low-throughput techniques such as qRT-PCR to other (older) high-throughput techniques such as microarrays, and the measurements from different techniques are often not directly comparable. How to use the information from these qualitative marker genes to assign cells to known cell types? Recently three methods have been developed: SCINA [16], Garnett [17], and CellAssign [18]. These methods are sometimes referred to as “semi-supervised” methods, as they only use the identity of marker genes, as opposed to methods that rely on a reference dataset, which are referred to as “supervised” methods.

SCINA uses a Gaussian mixture model to describe the expression data and introduces a constraint that marker genes should have higher mean expression levels in their corresponding cell types. CellAssign uses a Bayesian probabilistic model that includes a dedicated factor to describe the over-expression of marker genes. Garnett first selects a set of “representative” cells for each known cell type, defined as cells that express highly on all marker genes of the cell type, trains a generalized-linear-model-based classifier using merely these representative cells, and then applies this classifier to other cells to determine their labels.

In this paper, we propose another “semi-supervised” method for cell-type assignment/calling. That is, our method completes the same task as SCINA, Garnett, and CellAssign: assigning cells into known cell types using the scRNA-seq expression data in hand and a list of marker genes for each known cell type. Again, marker genes for a cell type are genes that are over-expressed (i.e., have higher expression levels) in that cell type, but their exact expression levels are not assumed known, and no reference dataset is used. Inspired by the parcel sorter, which is used by postal services to distribute mails to different paths/targets according to features of the parcel such as the zip code and the package size, we name our method single-cell sorter, or scSorter for short, as it assigns single cells to different predefined cell types according to the over-expression of marker genes.

By allowing flexibility in marker gene expression, borrowing information from non-marker genes, and using a two-step procedure, scSorter shows much superior power over existing methods on both simulated and real datasets. In all the simulated datasets, scSorter reduces the error rate significantly across all simulation scenarios. In the real datasets, on average, scSorter reduces the error rate of competitors by 58 % or more.

## Results

### Description of simulated data

We simulated data under three scenarios. In the first scenario, we simulated ten cell types and the marker genes for each of these ten cell types were given. In this case, there were no unknown cell types in the expression data, and all known cell types appeared in the (scRNA-seq) expression data. This was the simplest scenario; we made things more challenging in the second and third scenarios. In the second scenario, the marker genes for ten cell types were given but some of these ten cell types did not appear in the expression data. In the third scenario, there were ten cell types in the expression data but the marker genes of some of these ten cell types were unknown and thus cells from these cell types should be assigned to “unknown.” It is worth noting that in scenarios 1 and 2, although no cell should be assigned to the unknown cell type as all cell types in the expression data were known, this information was not passed to computational algorithms assigning cell types and thus some cells might still be labeled as unknown. This was to mimic the situation in real data analysis.

Simulated datasets were generated using Splatter [19] with the extensions developed in study [18] to deal with the extreme log fold change they observed in real data, and with further corrections to better describe the excessive zeros observed in real data. Each simulated dataset consisted of 5000 cells from 10 cell types. Each cell type contained two to five marker genes. In scenario 2, the expression data from some cell types (one, two, or three) were removed, and in scenario 3, the identities of marker genes from some cell types (one, two, or three) were removed. All simulations were repeated 50 times. More details about how we generated the simulated data are provided in Additional file 1.

### Description of real datasets

We used five real datasets, and we referred them by the first author of the corresponding paper or by the content of the data. All the real datasets were normalized by LogNormalize method from Seurat (version 3.1.0) prior to analysis. The marker genes used for all the datasets are listed in Additional file 1. Highly variable genes were chosen using the “vst” method in the Seurat package.

*Rosenberg data*: This data was created by Rosenberg et al. [20] using the SPLiT-seq protocol to analyze cells from the mouse brain and spinal cord. It profiled 27,096 non-neuronal cells from different cell types: Oligo (4,294 cells), OPC (5,793 cells), Immune (621 cells), Vascular (659 cells), VLMC (1,474 cells), Astrocyte (13,481 cells), Ependyma (518 cells), and OEC (256 cells). We used the cell type definition as well as marker genes defined in the original study [20].

*TM Pancreas data*: This data was a mouse atlas created by the Tabula Muris Consortium [21]. The cells were sorted using fluorescence-activated cell sorting (FACS) and sequenced by Smart-seq2 protocol. 1564 cells with valid cell type annotation from a pancreas tissue were used for our analysis. They included cells from Pancreatic A (390 cells), Pancreatic B (449 cells), Pancreatic D (140 cells), Pancreatic PP (73 cells), Pancreatic Acinar (182 cells), Pancreatic Ductal (161 cells), Pancreatic Stellate (49 cells), Endothelial (66 cells), and Immune (54 cells). Marker genes for these cell types were extracted from the original study [21].

*MCA lung data*: Using Microwell-seq protocol, this data was a mouse atlas created by Han et al. [22]. Lung data from this atlas was selected and used by Garnett [17] to illustrate its performance. 6940 lung cells with cell type annotation came from alveolar (2215 cells), B (728 cells), ciliated (317 cells), endothelial (321 cells), fibroblasts (412 cells), granulocytes (349 cells), macrophage (772 cells), dendritic (887 cells), natural killer (NK, 275 cells), and T (664 cells). Marker genes for these cell types were extracted from the original study [22].

*PBMC data*: Using the 10X Chromium platform (Cell Ranger version 1.1.0), this data was created by Zheng et al. [23]. It contained 94,655 peripheral blood mononuclear cells (PBMCs) from the following cell types: T (64,341 cells), B (10,085 cells), CD34+ (9232 cells), NK (8385 cells), and monocytes (2612 cells). This dataset was used by Garnett to illustrate its performance [17], with the difference that Garnett considered two subtypes of T cells, CD4+ and CD8+, to specifically demonstrate its performance under cell type hierarchy. The marker-gene list we used for the five cell types was extracted from the Garnett paper. (For T cells, the marker genes are *CD3D*, *CD3E*, and *CD3G*.)

*CBMC data*: This data was created by Stoeckius et al. [24] using CITE-seq on the 10x platform. It contained a mixture of 8617 human and mouse cells with the mouse cells (about 5% of the total cells) used as negative controls for the protein measurements. Here, we used the 7654 human cord blood mononuclear cells (CBMC) with cell types determined by antibody-derived tags (ADTs) from the following cell types: CD4 T cells (3065 cells), CD8 T cells (293 cells), B (364 cells), CD34+ (226 cells), NK (1205 cells), and monocytes (2501 cells). The marker-gene list for these six cell types were summarized from previous studies [17, 25, 26].

### The motivation and novelties of scSorter

**Fig. 1:**
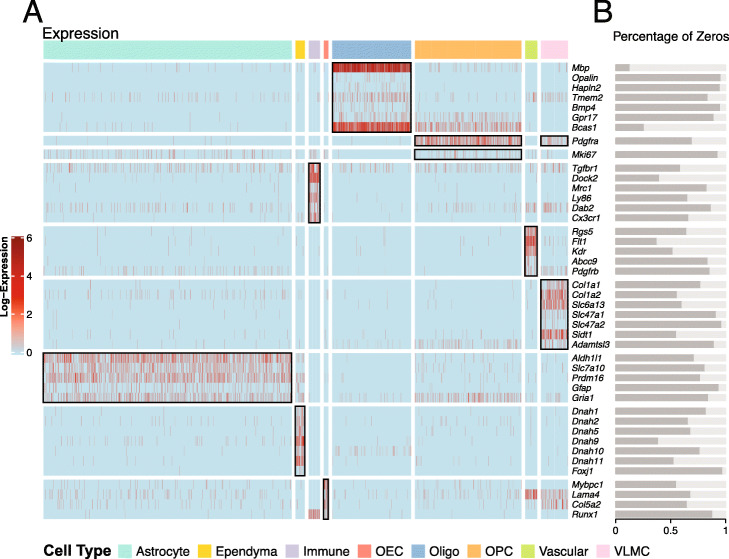
Visualization of Rosenberg data. **a** Expression of cells from different cell types on marker genes. Columns are cells and rows are marker genes. Cells are grouped according to the (true) cell types that they belong to, with the colorful bar on the top of the expression matrix giving the cell type identities, and the marker genes are grouped according to the cell types that they correspond to. The white vertical and horizontal gaps separate cells from different types and marker genes corresponding to different cell types. The black boxes indicate the correspondences of cell types and marker genes. **b** The bars show the percentages of cells that have zero expression in the corresponding cell types, for the marker genes

scSorter is a very different method from SCINA, Garnett, and CellAssign. It is initiated from an observation about scRNA-seq data: marker genes, even those well-documented ones, may not, and actually typically do not, have higher expression in all the cells in the corresponding cell types. This important observation was first raised and discussed in a recent manuscript [13], and it is validated on the real datasets we investigated. Take Rosenberg data as an example. Figure 1a shows the expression of marker genes in cells from different cell types. Cells are grouped according to the (true) cell types they belong to, and the black boxes indicate the correspondences of cell types and marker genes. For example, for the Oligo cells, the expression of genes *Mbp* to *Bcas1* are surrounded with a black box, indicating that these genes are marker genes for cell type Oligo.

This plot clearly shows that most marker genes do not consistently have high expression levels across all cells in the corresponding cell type. For example, for the seven marker genes for cell type Oligo, the proportions of the Oligo cells that have zero expression of these genes are 13% (*Mbp*), 96% (*Opalin*), 95% (*Hapln2*), 84% (*Tmem*2), 95% (*Bmp4*), 89% (*Gpr17*), and 26% (*Bcas1*). We see that all of them are missed in expression in some proportions of the cells, and most of them have zero expression in over 80% of the cells. This is biologically meaningful, as the Oligo cells form a lineage, and these marker genes are known to express only at certain intermediate stages of oligodendrocyte development [20]. For each marker gene, Fig. 1b shows the percentage of cells where its expression is zero in its corresponding cell type. On average, this percentage is 72%.

The missing of expression of marker genes is actually quite common in real datasets. It is observed in all the five real datasets we use, and in four out of the five real datasets, over half of the marker genes have zero expression in the majority of cells of the corresponding cell types (i.e., in the cell types where they are marker genes).

There are quite a few possible causes of this phenomenon. First, some marker genes are expected, biologically, to be expressed at high levels only in a proportion, but not all, of the cells in the corresponding cell type. These genes are expected, biologically, to be expressed consistently at low levels in other cell types, and thus, they are still valid markers. An example is the marker genes for the Oligo cell type, which we just discussed. A second cause can be the high heterogeneity of cell expressions. Cells, even from the same “cell type,” may have large biological variations in their expression levels. A third cause can be the high noise level in scRNA-seq data. That is, even if two cells have exactly the same expression of a gene, the observed expression measurements from a scRNA-seq experiment can still be quite different due to large systematic biases/variations, e.g., the so-called dropout events [27]. A fourth cause can be that the a priori knowledge about the identities of some marker genes may be inaccurate or inapplicable to the new dataset.

To accommodate such phenomenon, scSorter proposes two strategies. First, it combines the expression of marker genes and other genes together for clustering. The idea is cells from the same/different cell type(s) are likely to have similar/different expression profiles, and this similarity/difference is not only on marker genes but also on some other genes, particularly so-called highly variable genes, which are known to be highly informative in clustering and thus used by most clustering algorithms developed for single-cell data (see, e.g., [4, 5, 28, 29]). For simplicity, in this paper, we call highly variable genes that are not marker genes “non-marker genes” or “other genes.” Considering that the number of non-marker genes is typically much larger than the number of marker genes, completely ignoring information from them can be a big waste of data. Thus, scSorter tries to borrow information from non-marker genes: when marker genes have a lot of zero expression, other genes may take over the role and decide the assignment; even when marker genes are expressed, other genes may still contribute and help to verify or adjust the assignment. Among the three existing methods, SCINA and CellAssign only use the expression of marker genes, and Garnett makes use of non-marker genes in the second step, i.e., building a classifier based on the “representative” cells. However, Garnett does not use non-marker genes in the first step, i.e., searching for the representative cells. scSorter uses non-marker genes in a more persistent and explicit way. It proposes an optimization problem where the target function is an explicit combination of marker gene expression and non-marker gene expression. Thus, its solution is always a joint effort of both of them.

The second strategy that scSorter proposes is to explicitly allow marker genes to express at a low level in some cells from their corresponding cell types. Each marker gene in each cell can “freely choose” to express at a base level (i.e., the expression level of this gene in cells that do not belong to this gene’s corresponding cell type) or an elevated level (i.e., a level that is higher than the base level). This choice is fully automatic, realized by solving a constrained optimization problem.

By allowing flexibility in marker gene expression and borrowing information from non-marker genes, scSorter tolerates marker genes’ inconsistency in over-expression and uses other genes to assist clustering.

Another major concern in cell type assignment is the existence of “unknown” genes. That is, some cells may not belong to any of the known cell types and need to be labeled as unknown. However, different from cells in any particular known cell type, these unknown cells typically consist of cells from many unknown cell types, which can be very different from each other, and thus they do not “cluster together.” scSorter circumvents this difficulty by using a novel two-step approach. Suppose there are *K* known cell types with marker gene information. In the first step, scSorter proposes and solves a constrained optimization problem that clusters all cells into *K* clusters. Each cluster given by the first step is expected to contain both cells that are from a known cell type and unknown cells that have expression profiles more similar to this known cell type than the other known cell types. These two categories of cells are then separated in the second step by scrutinizing into their expressions on both marker genes and non-marker genes. A formal statistical test is proposed for the separation. We can call the first step “clustering” and the second step “unknown-cell calling.” A detailed description of the scSorter algorithm is given in the “Methods” section.

### Application of scSorter and four other algorithms

scSorter was applied to all the simulated datasets and real datasets with the same simple setting of a common weight parameter *w*_0_=2.0 (introduced in the “Methods” section) and all other parameters automatically determined by the algorithm.

The performance of three other approaches, SCINA, Garnett, and CellAssign (details about how we used these three approaches are available in Additional file 1), was reported and compared with scSorter.

All these three existing approaches, as well as scSorter, are based on their own dedicated models/strategies that integrate the over-expression of marker genes into the clustering procedure. One might, however, proposes a more straightforward two-step approach that first uses a regular (without considering marker gene information) clustering algorithm to cluster cells and then assigns the most parsimonious cell type to each cluster based on the average marker-gene expression. We implemented such an approach. In the first step, SC3 [5] was used for clustering (the reason why we used SC3 instead of Seurat is discussed in Additional file 1), and in the second step, the Pearson correlation coefficient was used for measuring parsimony. See Additional file 1 for a detailed description of this method. We call this method “SC3+correlation”. It is worth noting that SC3, which is a completely unsupervised approach for clustering, alone cannot be used to assign cells to known cell types, and this is also why we do not call this straightforward two-step approach “SC3”. The performance of SC3+correlation was also reported and compared with the other four methods.

### Measurement of performance

The performance of scSorter, as well as SCINA, Garnett, CellAssign, and SC3+correlation, was measured by misclassification rate, defined as the proportion of cells that were not classified into the correct cell types. It equals 1 minus classification accuracy, and a lower value means better performance. Misclassification rate is typically used for (supervised) classification problems, but it was suitable to be used for our (unsupervised) clustering problem as the true cell types of all our simulated and real datasets were known, and we not only wanted to put cells coming from the same cluster into *a* cluster, but also into a *specific* cluster which has the matched marker genes.

### Performance on simulated data

**Fig. 2:**
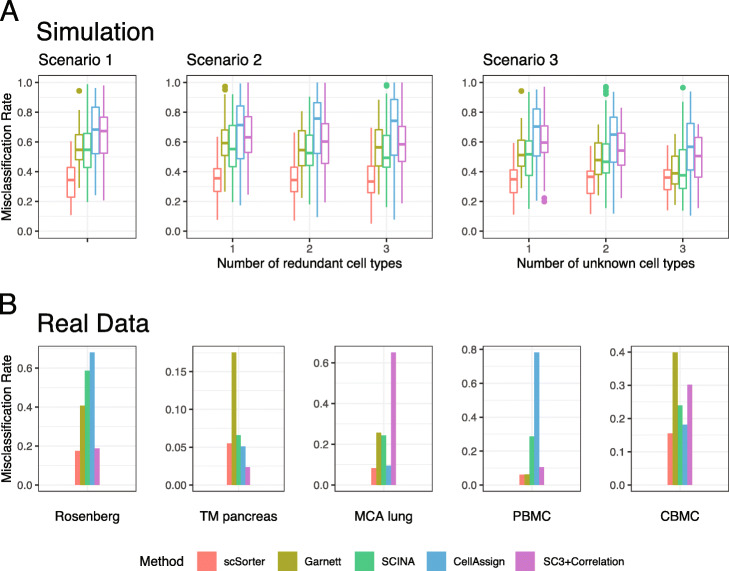
The performance of scSorter and four other methods on simulated and real datasets. **a** The performance of the five methods on simulated data of three scenarios. In the first scenario, we simulated ten cell types and the marker genes for each of these ten cell types were given. In this case, there were no “unknown” cell types in the expression data, and all known cell types appeared in the (scRNA-seq) expression data. This was the simplest scenario. In the second scenario, the marker genes for ten cell types were given but some of these ten cell types did not appear in the expression data. In the third scenario, there were ten cell types in the expression data but the marker genes of some of these ten cell types were unknown and thus cells from these cell types should be assigned to “unknown.” **b** The performance of the five methods on five real datasets

The misclassification rates of the methods under comparison are shown in Fig. 2a. The results are consistent across all simulation scenarios/settings: scSorter gave considerably lower misclassification rates than all the other four methods. On average, the misclassification rate of scSorter was 33.1% lower than that of SCINA, 34.1% lower than that of Garnett, 46.5% lower than that of CellAssign, and 41.3% lower than that of SC3+correlation.

### Performance on real datasets

The misclassification rates of the five methods on five real datasets are given in Fig. 2b. scSorter achieved the lowest misclassification rate in almost all the datasets, with the only exception on TM Pancreas data, where CellAssign and SC3+correlation gave lower misclassification rates of 0.0512 and 0.0237, respectively, compared to 0.0550 from scSorter. On average across the five datasets, the misclassification rate of scSorter is 62.7% lower than that of SCINA, 59.3% lower than that of Garnett, 70.4% lower than that of CellAssign, and 58.2% lower than that of SC3+correlation. Moreover, scSorter was the only method that achieved misclassification rate less than 20% in all the five datasets, while all other methods struggled in giving highly accurate results in at least one dataset: SCINA gave wrong assignments in more than 50% of cells in Rosenberg data, Garnett gave wrong assignments in around 40% of cells in Rosenberg data and CBMC data, CellAssign gave wrong assignments in more than 60% of cells in Rosenberg data and PBMC data, and SC3+correlation gave wrong assignments in more than 60% of cells in MCA lung data.

**Fig. 3:**
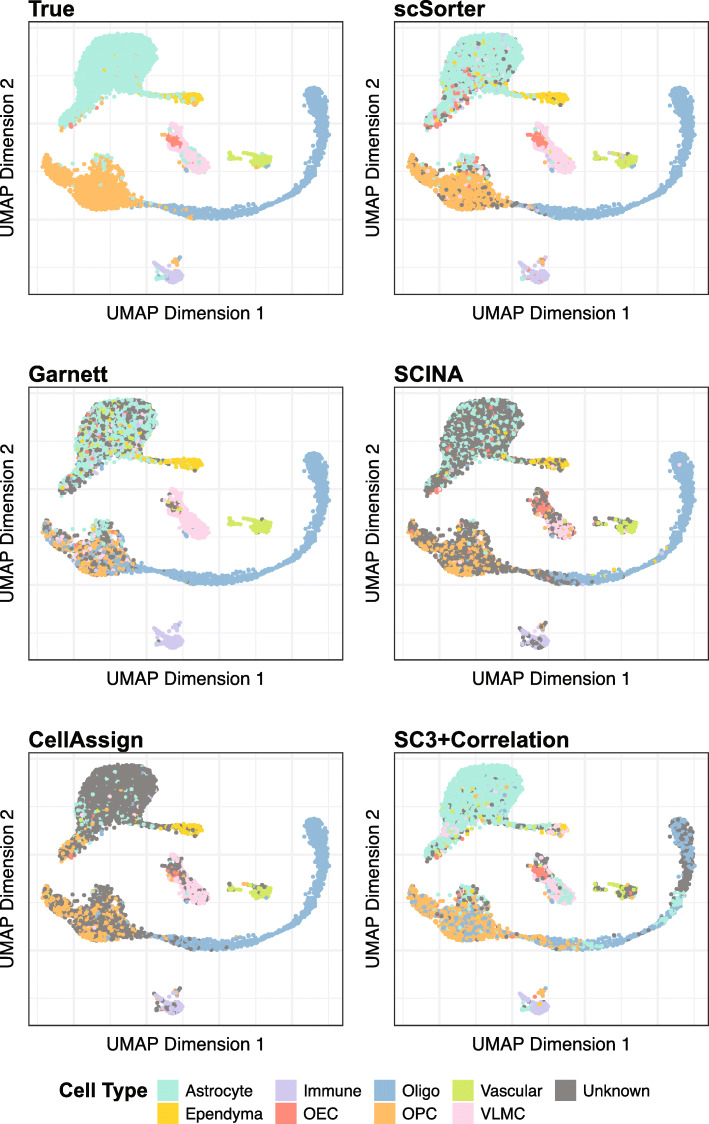
UMAP plots of Rosenberg data. The first panel is colored by true cell types. The other five panels are colored by cell types assigned by the corresponding methods

We take a closer look at the results on Rosenberg dataset. As shown in Fig. 1a and b, averagely marker genes have zero expression in 72% of the cells of their corresponding types. Figure 3 shows the detailed cell-type calling results of scSorter and the other four methods, visualized on the two-dimensional space produced by UMAP [30]. Take two cell types, Astrocyte and OPC, as examples. Compared to scSorter, which called the cell types correctly for most cells in both cell types, SCINA, Garnett, and CellAssign failed to make an assignment (i.e., assigned to “Unknown”) for a significant proportion of cells. The higher calling rate of scSorter on these two cell types should be easy to understand from Fig. 1b, as every marker gene from these cell types have zero expression in more than 60% of cells in the corresponding cell type. In this case, while scSorter might efficiently utilize expression of non-marker genes to help make an assignment, SCINA, Garnett, and CellAssign might just give up. We also found Garnett mistakenly assigned a large proportion of cells lying on the rightmost tip of the OPC cluster into the Oligo cell type, which scSorter did not. This indicates that even when some marker genes are expressed (especially, *Mbp* and *Bcas1* for Oligo, as shown in Fig. 1), scSorter may be able to efficiently use the expression of non-marker genes to adjust or correct the decision made by marker genes; this could be the case if the evidence from marker genes are weak but the evidence from non-marker genes are of high confidence. A detailed discussion regarding this is available in the “The OPC and Oligo cell types in Rosenberg data” section of Additional file 1.

TM pancreas dataset is somewhat different. As Additional file 1: Figure S1A&B shows, only seven out of 25 marker genes have zero expression in more than 10% of the cells in their corresponding cell types. That means that only relying on the expression of marker genes might be enough to give accurate cell type calling. Indeed, SCINA and CellAssign, both of which use only marker gene expression, performed comparably to scSorter (misclassification rates 0.0659 from SCINA and 0.0512 from CellAssign, compared to 0.0550 from scSorter). Garnett somehow performed much worse (misclassification rate 0.1753). Additional file 1: Figure S2 shows the detailed cell-type calling results visualized by UMAP.

The expression of marker genes in MCA lung dataset is highly inconsistent. Not only 74% of all marker genes have zero expression in over a half of cells of their corresponding types, as shown in Additional file 1: Figure S3B, but also a non-negligible proportion of marker genes are highly expressed in cells out of the cell types in which they are specified. For example, *Cd74* (to highlight, we colored this gene name red in Additional file 1: Figure S3A) is the marker gene of both dendritic cells and macrophage cells, but it is also highly expressed in most B cells and a majority of alveolar cells. As another example, *H2-ebt*, *H2-aa*, and *H2-ab1* (all highlighted in Additional file 1: Figure S3A) are marker genes of dendritic cells, but they are also highly expressed in a majority of B cells and a significant proportion of macrophage cells. On this data, scSorter gave much superior performance over SCINA and Garnett, reducing their misclassification rates by about two thirds (scSorter 0.0831 vs. SCINA 0.2431 vs. Garnett 0.2574). Additional file 1: Figure S4 shows the detailed cell-type calling results visualized by UMAP. Looking more closely, we found that while scSorter correctly assigned 97.8% of alveolar cells, SCINA and Garnett assigned only 91.5% and 75.1%, respectively. SCINA also failed to cluster significant proportions of endothelial cells and dendritic cells, and Garnett failed in significant proportions of dendritic cells and B cells. The result on this dataset indicates that scSorter is capable of dealing with challenging datasets where the marker genes have highly variable and non-specific expression. SC3+correlation failed miserably on this dataset by failing to assign over 60% of cells. Actually, among the ten known cell types, it only called three cell types: granulocytes, fibroblasts, and alveolar. This could reflect a problem of such straightforward two-step approaches: the first step, clustering, does not take advantage of the list of known marker genes. Such an “unguided” first step is less likely to identify clusters that reflect the true cell types (i.e., cell clusters that users desire), and if the first step fails, the whole algorithm will fail almost inevitably.

Marker genes in PBMC data also tend to have zero expression in their corresponding cell types, as shown in Additional file 1: Figure S5A&B. The average proportion is about 67%. This could be the reason that SCINA, a method that merely relies on the expression of marker genes, performed much worse than scSorter and Garnett. Another algorithm, CellAssign, performed even worse than SCINA. The reason could be that this data has highly imbalanced numbers of cells in different cell types, and CellAssign tends to fail on such datasets (see Additional file 1 for detail). The marginally better performance of scSorter compared to Garnett was largely due to its success in assigning a larger proportion (98.0% vs. 94.9%) of T cells to the right cell type instead of labeling them as unknown. Additional file 1: Figure S6 shows the detailed cell-type calling results visualized by UMAP.

CBMC data shares cell types with PBMC data. The average proportion of zero expression observed on marker genes in their corresponding cell types, as shown in Additional file 1: Figure S7, is about 55%, lower than that of PBMC data. scSorter performed the best among all methods under comparison. The improved performance of the other methods on this data, compared to PBMC data, could be due to the lower proportion of zeros on marker genes and to the more balanced sizes of different cell types. Additional file 1: Figure S8 shows the detailed results for cell-type calling visualized by UMAP.

## Discussion

**Fig. 4:**
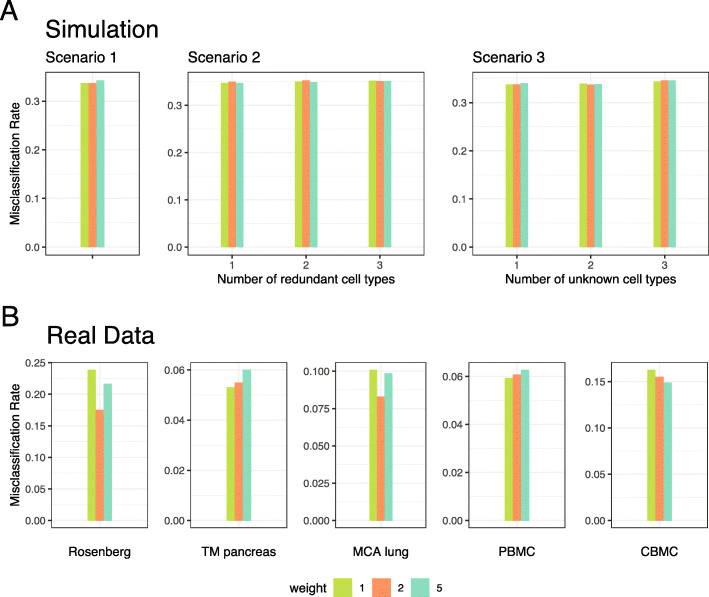
The performance of scSorter with three choices (1.0, 2.0, 5.0) of marker gene weight. **a** The performance of scSorter on simulated data with three choices of marker gene weight. **b** The performance of scSorter on five real datasets with three choices of marker gene weight

scSorter is easy to tune up. Practically, only one parameter, the weight(s) for marker genes (*w*_*ik*_ in Eq. 1) needs to be specified manually. As we have discussed, larger *w*_*ik*_ values reflect more confidence in the correctness/applicability of marker genes. This way, users may be easier to decide whether to add marker genes they are not very sure about: just add them with smaller weights. When no information about confidence levels of marker genes is available, simply putting a common weight is suggested. In all the calculations of this paper, we used a common weight 2.0, meaning that the contribution of marker genes in deciding the assignments is roughly two times that of non-marker genes. To study the sensitivity of scSorter to different values of this common weight, we ran scSorter on all simulated data and real datasets with three different weights: 1.0, 2.0, and 5.0. Misclassification rates under different weights are shown in Fig. 4a and b. We see that the weight parameter has a minor to moderate effect on the performance. While different datasets have different optimum weights, 2.0 is a decent choice on all our simulated data and real datasets. We set 2.0 as the default value for our R package, which is named scSorter and freely available on CRAN.

scSorter has a “flat” clustering scheme. While it can surely be used for data with cell type hierarchy, it does not particularly take advantage of the hierarchy information. In the “Results” section, when we applied it on PBMC data, we combined the two subtypes of T cells, CD4+ and CD8+. If the CD4+ and CD8+ were treated as two cell types, the misclassification rate for scSorter, Garnett, SCINA, CellAssign, and SC3+correlation would instead be 0.1302, 0.0819, 0.3157, 0.6739, and 0.5137, respectively. We see that while scSorter still significantly outperformed SCINA, CellAssign, and SC3+correlation, Garnett outperformed scSorter. The outperformance of Garnett over scSorter, however, was not consistent across all cell types: while scSorter misclassified 13.0% of CD4+ T cells and 11.7% of CD8+ T cells, Garnett misclassified 1.7% of CD4+ T cells and 15.8% CD8+ T cells.

In our real data analysis, we used the “vst” method in Seurat to choose highly variable genes. We also tried two other methods in Seurat, “mean-variance-plot” and “dispersion”, and found that the misclassification rate (given in Additional file 1: Table S1) of scSorter barely changed, suggesting that the performance of scSorter is rather stable with different choices of highly variable genes.

## Conclusions

We have presented scSorter, an algorithm that assigns cells to known cell types based on the identities of marker genes. scSorter is based on the observation that marker genes, which are expected to express in higher levels in the corresponding cell types, may in practice express at a very low level in many of those cells. scSorter takes full use of such feature and allows cells to express either at an elevated level or a base level, without a direct penalty. Another important characteristic of scSorter is the explicit utilization of expression profiles of both marker genes and non-marker genes. By combining them, scSorter is able to not only call more cells but also make more accurate calls. The superior performance of scSorter has been demonstrated in both simulated and real datasets. Its improvement over existing methods was substantial.

## Methods

### Notations and settings

Suppose there are *K* known cell types. For each cell type, there are a certain number of marker genes, and we have *g* marker genes in total, that is, *g* genes are markers for at least one cell type. Except for these *g* genes, we also consider *h* non-marker genes. That is, we consider *g*+*h* genes in total.

Suppose we have *N* cells, and then the expression data we consider can be written as a matrix ***X*** of dimension (*g*+*h*)×*N*, with element *X*_*ij*_ being the expression of gene *i* in cell *j*, *i*=1,…,*g*+*h* and *j*=1,…,*N*. Without loss of generality, we assume the first *g* genes are marker genes, and the remaining *h* genes are highly variable genes. We also assume the gene expression has been properly normalized for the library size and has been properly transformed (e.g., log-transformation) to stabilize the variance.

Suppose the prior knowledge of marker genes (i.e., which gene is the marker gene for which cell type) is summarized as an indicator matrix ***Γ*** of dimension *g*×*K*, with *γ*_*ik*_=1 denoting that gene *i* is a marker gene of cell type *k* and *γ*_*ik*_=0 otherwise.

To record the results of clustering, we let *C*_*k*_ be the index set of cells belonging to cell type (cluster) *k*. That is, *j*∈*C*_*k*_ means that cell *j* is assigned to cell type *k*. We let the number of cells in *C*_*k*_ be *n*_*k*_.

### Step I: Clustering by solving a novel optimization problem

scSorter proposes the following optimization problem: find ***C***={*C*_*k*_}_*k*=1,...,*K*_,***μ***={*μ*_*i*_}_*i*=1,…,*g*_∪{*μ*_*ik*_}_*i*=*g*+1,...,*g*+*h*,*k*=1,...,*K*_, and ***δ***={*δ*_*ik*_}_*i*=1,...,*g*;*k*=1,...,*K*_ that

1 $$\begin{array}{*{20}l} {}\text{minimize} \quad &\sum_{i=1}^{g}\sum_{k=1}^{K}\sum_{j \in C_{k}}w_{ik}\left [(x_{ij}-\mu_{i})^{2}I_{\gamma_{ik}=0}+\min((x_{ij}-\mu_{i}-\delta_{ik})^{2},(x_{ij}-\mu_{i})^{2}) I_{\gamma_{ik}=1}\right]  \\ + &\sum_{i=g+1}^{g+h}\sum_{k=1}^{K}\sum_{j \in C_{k}}(x_{ij}-\mu_{ik})^{2}  \end{array} $$

2 $$\begin{array}{*{20}l} {}\text{subject to} \quad &\delta_{ik} \geq 0,\qquad i=1,\ldots,g; k=1,\ldots,K.  \end{array} $$

On the above, *w*_*ik*_ is a pre-specified weight (a positive constant) for marker gene *i* of known cell type *k*, *i*=1,…,*g*. A larger *w*_*ik*_ reflects more confidence in the role of gene *i* being a marker gene of cell type *k*. Based on the background biological knowledge, users can set proper *w*_*ik*_ values accordingly. When such knowledge is not available, we suggest simply choosing a constant value for all marker genes: *w*_*ik*_=*w*_0_·*h*/*g*. Considering that there are in total *g* marker genes and *h* non-marker genes, this choice means that the overall contribution of the marker genes to target function is *w*_0_ times of the non-marker genes. In this case, *w*_0_ reflects how much we want to rely on the marker genes in determining the cell types. In all data analysis of this paper, we use this simple choice with *w*_0_=2.

In Eq. 1, for a non-marker gene (i.e., *g*+1≤*i*≤*g*+*h*), the cost of assigning cell *j* to cluster *k* is defined as (*x*_*ij*_−*μ*_*ik*_)^2^. It is actually easy to show (see Additional file 1 for details) that the solution for *μ*_*ik*_ is the centroid (mean expression level) of cluster *k*. Thus, this cost on each non-marker gene $\sum _{k=1}^{K}\sum _{j \in C_{k}}(x_{ij}-\mu _{ik})^{2}$ is the sum of distances from each cell to the corresponding centroid, which is exactly the same as the regular K-means algorithm.

On the other hand, for a marker gene (i.e., *i*≤*g*), the cost is defined in a different way. In Eq. 1, *I*_condition_ is the “indicator function,” which equals 1 when the condition holds and equals 0 otherwise, and min(·,·) means the smaller of the two items in the parentheses. For gene *i* that is a marker gene for cell type *k*, its expression is assumed to be *μ*_*i*_ for cells not in cell type *k* (i.e., *γ*_*ik*_=0), and assumed to be either *μ*_*i*_+*δ*_*ik*_ or *μ*_*i*_, whichever is closer to *x*_*ij*_, for cells in cell type *k* (*γ*_*ik*_=1). Considering that *μ*_*i*_+*δ*_*ik*_≥*μ*_*i*_, which is guaranteed by the constraint (i.e., Eq. 2), we call *μ*_*i*_+*δ*_*ik*_ the elevated (expression) level, and *μ*_*i*_ the base (expression) level. By letting the expression of a marker gene in its corresponding cell type to freely choose from the elevated and the base levels, we accommodate the observation that marker genes may not be over-expressed in a significant proportion of cells of the corresponding type. Their expression may actually be at the base level, i.e., similar to their expression in other cell types.

### The solution to step I

Although having many unique features, our target function, Eq. 1, looks similar to that of the classic K-means clustering algorithm. And just like K-means, overall our target function is a combinatorial optimization problem, and there is no easy algorithm that guarantees to find the global optimum. Meanwhile, we have found that the idea of solving K-means, alternatively optimizing the cluster assignments and the centriods, works highly efficiently for our optimization problem in finding a local optimum. The alternative optimization in scSorter is as follows.

1. Keeping ***μ*** and ***δ*** unchanged, update ***C***. This can be done by assigning cell *j* to the cluster that gives the smallest cost:
  $$ \sum_{i=1}^{g}w_{ik}\left [(x_{ij}-\mu_{i})^{2}I_{\gamma_{ik}=0}+\min((x_{ij}-\mu_{i}-\delta_{ik})^{2},(x_{ij}-\mu_{i})^{2}) I_{\gamma_{ik}=1}\right] + \sum_{i=g+1}^{g+h}(x_{ij}-\mu_{ik})^{2}. $$
2. Keeping ***C*** unchanged, update ***μ*** and ***δ***. Note that our target function is separable on *i* (i.e., genes), and thus solving ***μ*** and ***δ*** can be done by solving them for each gene independently.
  For non-marker genes, *μ*_*ik*_ has a simple closed-form solution: $\hat {\mu }_{ik}=\frac {1}{n_{k}}\sum _{j \in C_{k}}x_{ij}$. For marker genes, *μ*_*i*_ and *δ*_*ik*_ do not have closed-form solutions, and we solve them by repetitively updating one given the other using the following two formulas
  $$\begin{array}{*{20}l} \hat{\delta}_{ik} = &\frac{\sum_{j \in C_{k}}(x_{ij}-\hat{\mu}_{i})I_{\gamma_{ik}=1}I_{x_{ij}>\hat{\mu}_{i}+\hat{\delta}_{ik}/2}}{\sum_{j \in C_{k}}I_{\gamma_{ik}=1}I_{x_{ij}>\hat{\mu}_{i}+\hat{\delta}_{ik}/2}}\\ \hat{\mu}_{i} = &\frac{\sum_{k = 1}^{K} \sum_{j \in C_{k}} w_{ik} x_{ij} - \sum_{k = 1}^{K} \sum_{j \in C_{k}} w_{ik} \hat{\delta}_{ik}I_{\gamma_{ik}=1}I_{x_{ij}>\hat{\mu}_{i}+\hat{\delta}_{ik}/2}}{\sum_{k = 1}^{K} \sum_{j \in C_{k}} w_{ik}} \end{array} $$
  until convergence.

Detailed derivations of the formulas are given in Additional file 1. With these formulas, scSorter initializes ***C*** by randomly assigning cells to cell types, and then it alternatively updates {***μ***,***δ***} and ***C*** until the cell type assignment ***C*** does not change, indicating convergence to a (local) optimum.

### Step II: Unknown cell-type calling

In step I, all cells are forced to be clustered into *K* clusters. In another word, cells that are actually not from any of the known cell types will still be forced to join one of the clusters. In this case, an unknown cell may be clustered into a cluster, say cluster 1, not because it is actually from the known cell type 1, but because comparing to other clusters, it is (may be just slightly) closer to (the centroid of) cluster 1. This cell may actually not be very similar to cell type 1, but as far as it is more similar to cell type 1 compared to other known cell types, assigning it to cluster 1 would still be the best choice in step I. Our task in step II, is to disengage it from cell type 1, which is done by testing whether its similarity to cell type 1 is sufficiently high.

Given the solution to our optimization problem in step I, for marker gene *i* for the *k*’th known cell type, its expression in cell *j*, which has been clustered into cluster *k* in step I, is at the base level if min((*x*_*ij*_−*μ*_*i*_−*δ*_*ik*_)^2^,(*x*_*ij*_−*μ*_*i*_)^2^)=(*x*_*ij*_−*μ*_*i*_)^2^, or equivalently, *x*_*ij*_≤*μ*_*i*_+*δ*_*ik*_/2, and its expression is at the elevated level otherwise. Suppose *α*_*kj*_ proportion of the marker genes for cell type *k* are expressed at the elevated level in cell *j*, i.e., $\alpha _{kj} = \sum _{i=1}^{g} I_{\gamma _{ik} = 1} I_{x_{ij} > \mu _{i}+\delta _{ik}/2} / \sum _{i=1}^{g} I_{\gamma _{ik} = 1}$. We use *α*_*kj*_ to group cells in cluster *k*, i.e., *C*_*k*_, to two sets: (1) set *D*_*k*_; cells in this set have *α*_*kj*_>*α*_0_, where *α*_0_ is a pre-specified constant. (2) set *U*_*k*_; cells in this set have *α*_*kj*_≤*α*_0_. (We use *D* and *U* for these two sets as they are the preceding letters of “decided” and “undecided” respectively, for easy memory.) In another word, *D*_*k*_ contains cells that are clustered in cluster *k* and have at least a certain proportion of known marker genes expressed at an elevated level; scSorter will claim these cells to be from cell type *k* without further investigation. On the other hand, cells in *U*_*k*_ have a large proportion of marker genes (unexpectedly) expressed at the base level; scSorter will further screen these cells one by one and see whether each of them looks “similar enough” to cells in set *D*_*k*_, those that have already been believed to be truly from cell type *k*. This check relies on the expression of non-marker genes.

For *j*∈*U*_*k*_, define a z-score $z_{ij} = \frac {x_{ij} - \nu _{ik}}{\sigma _{ik}}$ for each non-marker genes *i*, *i*=*g*+1,…,*g*+*h*. Here $\nu _{ik} = \frac {1}{|D_{k}|}\sum _{j \in D_{k}}x_{ij}$ and $\sigma _{ik}=\sqrt {\frac {1}{|D_{k}|-1}\sum _{j \in D_{k}}(x_{ij}-\nu _{ik})^{2}}$, where |*D*_*k*_| denotes the number of items in set *D*_*k*_. Note that here both *ν*_*ik*_ and *σ*_*ik*_ are estimated using *D*_*k*_. Further, we define $d_{jk} = \sum _{i=g+1}^{g+h}z^{2}_{ij}$ and $\beta _{jk}=F_{\chi ^{2}_{h}}(d_{jk})$, where $F_{\chi ^{2}_{h}}$ is the cumulative distribution function of a *χ*^2^ distribution with degrees of freedom *h*. Thus, if this cell *j*, which is in *U*_*k*_, has similar expression profile to those cells in *D*_*k*_ on non-marker genes, *d*_*jk*_ should approximately follow a *χ*^2^ distribution with degrees of freedom *h*, and so *β*_*jk*_ should be uniformly distributed between 0 and 1. Otherwise, *d*_*jk*_ should be larger, and *β*_*jk*_ should be closer to 1. Accordingly, using a cutoff *θ*_*k*_, scSorter declares cells with *β*_*jk*_≤*θ*_*k*_ as in cell type *k* and cells with *β*_*jk*_>*θ*_*k*_ as in “unknown cell type.”

scSorter uses the following algorithm to automatically select *θ*_*k*_. Since cells in cluster *k* identified in step I are divided into set $D_{k}^{*} = D_{k} \cup (U_{k} \cap \{j; \beta _{jk} \le \theta _{k}\})$, which contains cells that are declared as in cell type *k*, and set $U_{k}^{*} = U_{k} \cap \{j; \beta _{jk} > \theta _{k}\}$, which contains cells declared as unknown, we search *θ*_*k*_ value that minimizes

3 $$ S_{k} = \sum_{i=g+1}^{g+h}\left[\sum_{j\in D_{k}^{*}}\left(x_{ij} - \bar{x}_{iD^{*}_{k}}\right)^{2} + \sum_{j\in U_{k}^{*}}\left(x_{ij} - \bar{x}_{iU^{*}_{k}}\right)^{2}\right]   $$

in the range of $\left [F_{\chi ^{2}_{h}}(h+\sqrt {2h}), 1\right ]$, where $h+\sqrt {2h}$ is the value that is one standard deviation on the right side of the mean of a *χ*^2^ distribution with degrees of freedom *h*. In Eq. 3, $\bar {x}_{iD^{*}_{k}}$ and $\bar {x}_{iU^{*}_{k}}$ are the average *x*_*ij*_ values in set $D^{*}_{k}$ and set $U^{*}_{k}$, respectively.

After step I (solving the optimization problem defined by Eqs. 1 and 2) and step II (unknown cell-type calling), all cells will be assigned, either to known cell types or as unknown. One problem, though, is that the assignment could be sub-optimum because the solution in step I could be only a local optimum. To mitigate this problem, we use the same strategy as that in K-means: we run the algorithm multiple times (ten times by default) with different initial values of ***C*** and choose the one that minimizes the final cost function, which we define as the cost function expressed in Eq. 1 with the second set of terms, $\sum _{i=g+1}^{g+h}\sum _{k=1}^{K}\sum _{j \in C_{k}}(x_{ij}-\mu _{ik})^{2}$, replaced by $\sum _{k=1}^{K} S_{k}$, where *S*_*k*_ is defined by Eq. 3. This strategy appears to work nicely (see Additional file 1: Table S2).

### Alternative approaches

Before settling down with the optimization problem (Eqs. 1 and 2) in step I, we have tried several other possible ways to formulate our clustering problem into an optimization problem. We have proposed the solution to each of them, written code to implement them, and run them on simulated data. They all have much inferior performance compared to the optimization problem we finally settled down with. Here we briefly describe these alternative forms of the optimization problem and give an explanation why they do not perform as well. The algorithms that solve each of these problems are given in Additional file 1. We think this content may be of interest to researchers who would like to further improve over scSorter, and it may also help to understand the reason why scSorter works well.

The first alternative is: find ***C***={*C*_*k*_}_*k*=1,...,*K*_ and ***μ***={*μ*_*ik*_}_*i*=1,...,*g*+*h*,*k*=1,...,*K*_ that

4 $$\begin{array}{*{20}l} \text{minimize} \qquad &\sum_{i=1}^{g}\sum_{k=1}^{K}\sum_{j \in C_{k}}w_{ik}(x_{ij}-\mu_{ik})^{2} + \sum_{i=g+1}^{g+h}\sum_{k=1}^{K}\sum_{j \in C_{k}}(x_{ij}-\mu_{ik})^{2}  \end{array} $$

5 $$\begin{array}{*{20}l} \text{subject to} \qquad &\left(\mu_{ik} - \frac{1}{N}\sum_{j=1}^{N}x_{ij}\right)I_{\gamma_{ik} = 1} \geq 0,\qquad i=1,\ldots,g; k=1,\ldots,K.  \end{array} $$

The second alternative is: find ***C***={*C*_*k*_}_*k*=1,...,*K*_ and ***μ***={*μ*_*ik*_}_*i*=1,...,*g*+*h*,*k*=1,...,*K*_ that

6 $$\begin{array}{*{20}l} \text{minimize} \qquad &\sum_{i=1}^{g}\sum_{k=1}^{K}\sum_{j \in C_{k}}w_{ik}(x_{ij}-\mu_{ik})^{2} + \sum_{i=g+1}^{g+h}\sum_{k=1}^{K}\sum_{j \in C_{k}}(x_{ij}-\mu_{ik})^{2}  \end{array} $$

7 $$\begin{array}{*{20}l} \text{subject to} \qquad &\min_{k \in \{k; I_{\gamma_{ik}=1}\}}\mu_{ik} \geq \max_{k \in \{k; I_{\gamma_{ik}=0}\}}\mu_{ik},\qquad i=1,\ldots,g.  \end{array} $$

The third alternative is: find ***C***={*C*_*k*_}_*k*=1,...,*K*_,***μ***={*μ*_*i*_}_*i*=1,…,*g*_∪{*μ*_*ik*_}_*i*=*g*+1,...,*g*+*h*,*k*=1,...,*K*_, and ***δ***={*δ*_*ik*_}_*i*=1,...,*g*;*k*=1,...,*K*_ that

8 $$\begin{array}{*{20}l} \text{minimize} \qquad &\sum_{i=1}^{g}\sum_{k=1}^{K}\sum_{j \in C_{k}}w_{ik}\left [(x_{ij}-\mu_{i})^{2}I_{\gamma_{ik}=0}+(x_{ij}-\mu_{i}-\delta_{ik})^{2}I_{\gamma_{ik}=1}\right]  \end{array} $$

9 $$\begin{array}{*{20}l} + &\sum_{i=g+1}^{g+h}\sum_{k=1}^{K}\sum_{j \in C_{k}}(x_{ij}-\mu_{ik})^{2} \end{array} $$

10 $$\begin{array}{*{20}l} \text{subject to} \qquad &\delta_{ik} \geq 0,\qquad i=1,\ldots,g; k=1,\ldots,K.  \end{array} $$

In the first alternative formation, note that when *γ*_*ik*_=0 (gene *i* is not a marker gene of cell type *k*), the constraints (Eq. 5) are automatically satisfied. Thus, the constraints only take effect on marker genes, which have to satisfy $\mu _{ik} \ge \frac {1}{N}\sum _{j=1}^{N}x_{ij}$, i.e., the representative expression of marker gene should be no less than the overall average.

In the first two alternative formations, a marker gene (i.e., *i*=1,…,*g*) has a different expression *μ*_*ik*_ in every cell type *k*. The constraints of the first formation (Eq. 5) are relatively weak: for a marker gene, although there is a constraint that its expression in cells of its corresponding cell type cannot be lower than its mean expression in all cells, there is no constraint on its expression in cells not of its corresponding cell type. As a result, the threshold for putting cells into a certain cell type is not high enough, and a large number of cells from other cell types, in which the marker genes of this cell type are also relatively highly expressed, may be incorrectly assigned to this type. The constraints in the second formation (Eq. 7) are much stronger. However, it is hard for every marker gene to satisfy these constraints, as its expression in cells of its corresponding cell type may, although high, not be higher than that in all the other clusters. As a result, the cells truly from a cell type, but in which the marker genes are not highly expressed, may be falsely excluded from this cell type. By using an elevated expression level *μ*_*i*_+*δ*_*ik*_ to cells in cell type *k* and a common base expression level *μ*_*i*_ to all other cells, the third alternative formation has constraints with strength stronger than the first alternative but weaker than the second. However, it still does not allow each marker gene to freely choose between an elevated level and a base level and thus does not fit the data as properly as scSorter.

## Supplementary Information

**Additional file 1** Supplementary Materials that include additional methods, results, and plots.

**Additional file 2** Review history.

## Data Availability

scSorter is implemented in R and freely available on CRAN (https://cran.r-project.org/) as an R package scSorter. [31] using GPL-3 license. The analysis code is deposited in zenodo with DOI:10.5281/zenodo.4459645 [32] using CC BY 4.0 license. The PBMC data is available from 10x Genomics website (https://support.10xgenomics.com/single-cell-gene-expression/datasets) under section *Single Cell 3’ Paper: Zheng et al. 2017*. The TM Pancreas data is available from figshare (https://figshare.com/articles/Single-cell_RNA-seq_data_from_Smart-seq2_sequencing_of_ FACS_sorted_cells_v2_/5829687). The MCA lung data is available from Gene Expression Omnibus (GEO) under accession number GSE108097. The Rosenberg data is available from GEO under accession number GSE110823.
