# Supplementary material for: scSorter: assigning cells to known cell types according to marker genes
Source: Genome Biol. 2021 Feb 22;22:69. doi: 10.1186/s13059-021-02281-7 (PMC7898451; doi:10.1186/s13059-021-02281-7)
Supplement: Supplementary file 1 — Additional file 1 Supplementary Materials that include additional methods, results, and plots. [file 13059_2021_2281_MOESM1_ESM.pdf]

Supplementary Materials for scSorter: assigning cells to known cell types according to marker genes

Hongyu Guo and Jun Li*

Department of Applied and Computational Mathematics and Statistics, University of Notre Dame, Notre Dame, IN 46556, USA

To whom correspondence should be addressed. Tel: +1 574 631 3429; Fax: +1 574 631 4822; Email: jun.li@nd.edu

Derivation of solutions for the scSorter algorithm

Let

$$S_{ij} = w_{ik}\left[(x_{ij}-\mu_i)^2 I_{\gamma_{ik}=0} + \min((x_{ij}-\mu_i-\delta_{ik})^2, (x_{ij}-\mu_i)^2) I_{\gamma_{ik}=1}\right], \tag{1}$$

then scSorter finds $\boldsymbol{C} = \{C_k\}_{k=1,\ldots,K}$, $\boldsymbol{\mu} = \{\mu_i\}_{i=1,\ldots,g} \cup \{\mu_{ik}\}_{i=g+1,\ldots,g+h,k=1,\ldots,K}$, and $\boldsymbol{\delta} = \{\delta_{ik}\}_{i=1,\ldots,g;k=1,\ldots,K}$ that

$$\text{minimize} \quad \sum_{i=1}^{g}\sum_{k=1}^{K}\sum_{j\in C_k} S_{ij} + \sum_{i=g+1}^{g+h}\sum_{k=1}^{K}\sum_{j\in C_k}(x_{ij}-\mu_{ik})^2 \tag{2}$$

$$\text{subject to} \quad \delta_{ik} \geq 0, \qquad i=1,\ldots,g; k=1,\ldots,K. \tag{3}$$

w_{ik} is a pre-specified weight (a positive constant) for marker gene i of known cell type k, $i = 1,\ldots,g$. γ_{ik} is an indicator with $\gamma_{ik} = 1$ denoting that gene i is a marker gene of cell type k and $\gamma_{ik} = 0$ otherwise.

An alternative optimization algorithm is used to solve scSorter as follows:

1. Keeping $\boldsymbol{\mu}$ and $\boldsymbol{\delta}$ unchanged, update $\boldsymbol{C}$. This can be done by assigning cell j to the

cluster that gives the smallest cost:

$$\sum_{i=1}^{g} S_{ij} + \sum_{i=g+1}^{g+h} (x_{ij} - \mu_{ik})^2.$$

2. Keeping $\boldsymbol{C}$ unchanged, update $\boldsymbol{\mu}$ and $\boldsymbol{\delta}$. Note that our cost function is separable on i (i.e. gene), and thus solving $\boldsymbol{\mu}$ and $\boldsymbol{\delta}$ can be done by solving them for each gene independently.

   For non-marker genes, μ_{ik} has a simple closed-form solution determined by:

$$\frac{\partial}{\partial \mu_{ik}} \sum_{j \in C_k} (x_{ij} - \mu_{ik})^2 = -2 \sum_{j \in C_k} (x_{ij} - \mu_{ik}) = 0, \tag{4}$$

   which gives

$$\hat{\mu}_{ik} = \frac{1}{n_k} \sum_{j \in C_k} x_{ij}. \tag{5}$$

   For marker genes, note that $\min((x_{ij} - \mu_i - \delta_{ik})^2, (x_{ij} - \mu_i)^2) = (x_{ij} - \mu_i - \delta_{ik})^2 I_{x_{ij} > \mu_i + \frac{\delta_{ik}}{2}} + (x_{ij} - \mu_i)^2 I_{x_{ij} \leq \mu_i + \frac{\delta_{ik}}{2}}$. This function is everywhere continuous but only piece-wise differentiable. The non-differentiable point satisfies $x_{ij} = \mu_i + \frac{\delta_{ik}}{2}$.

   We try to find δ_{ik} that minimizes $\sum_{j \in C_k} S_{ij}$. Again, this is a function that is everywhere continuous but only piece-wise differentiable, with n_k non-differentiable points that satisfy $x_{ij} = \mu_i + \frac{\delta_{ik}}{2}$ for $j \in C_k$. Thus, the minimum/maximum point should be at one of these non-differentiable points or at a differentiable point that satisfies

$$\frac{\partial}{\partial \delta_{ik}} \sum_{j \in C_k} S_{ij} = -2 \sum_{j \in C_k} w_{ik} \left[(x_{ij} - \mu_i - \delta_{ik}) I_{\gamma_{ik}=1} I_{x_{ij} > \mu_i + \frac{\delta_{ik}}{2}} \right] = 0,$$

   which gives

$$\hat{\delta}_{ik} = \frac{\sum_{j \in C_k} (x_{ij} - \hat{\mu}_i) I_{\gamma_{ik}=1} I_{x_{ij} > \hat{\mu}_i + \hat{\delta}_{ik}/2}}{\sum_{j \in C_k} I_{\gamma_{ik}=1} I_{x_{ij} > \hat{\mu}_i + \hat{\delta}_{ik}/2}}. \tag{6}$$

Actually, since at this point, we have

$$\frac{\partial^2}{\partial \delta_{ik}^2} \sum_{j \in C_k} S_{ij} = 2 \sum_{j \in C_k} w_{ik} I_{\gamma_{ik}=1} I_{x_{ij} > \mu_i + \frac{\delta_{ik}}{2}} > 0, \tag{7}$$

this $\hat{\delta}_{ik}$ is a local minimum point. We will use this $\hat{\delta}_{ik}$ for our iteration and ignore those non-differentiable points.

Similarly, we can try to find μ_i that minimizes $\sum_{k=1}^{K} \sum_{j \in C_k} S_{ij}$. Again, this is a function that is everywhere continuous but only piece-wise differentiable, with n possible non-differentiable points that satisfy $x_{ij} = \mu_i + \frac{\delta_{ik}}{2}$ for $j = 1, \ldots, n$. Thus, the minimum/maximum point should be at one of these non-differentiable points or at a differentiable point that satisfies

$$\begin{aligned}\frac{\partial}{\partial \mu_i} \sum_{k=1}^{K} \sum_{j \in C_k} S_{ij} =& -2 \sum_{k=1}^{K} \sum_{j \in C_k} w_{ik} [(x_{ij} - \mu_i) I_{\gamma_{ik}=0} + (x_{ij} - \mu_i - \delta_{ik}) I_{\gamma_{ik}=1} I_{x_{ij} > \mu_i + \frac{\delta_{ik}}{2}} \\ &+ (x_{ij} - \mu_i) I_{\gamma_{ik}=1} I_{x_{ij} \leq \mu_i + \frac{\delta_{ik}}{2}}] = 0,\end{aligned}$$

which gives

$$\hat{\mu}_i = \frac{\sum_{k=1}^{K} \sum_{j \in C_k} w_{ik} x_{ij} - \sum_{k=1}^{K} \sum_{j \in C_k} w_{ik} \hat{\delta}_{ik} I_{\gamma_{ik}=1} I_{x_{ij} > \hat{\mu}_i + \hat{\delta}_{ik}/2}}{\sum_{k=1}^{K} \sum_{j \in C_k} w_{ik}}. \tag{8}$$

Considering that

$$\begin{aligned}\frac{\partial^2}{\partial \mu_i^2} \sum_{k=1}^{K} \sum_{j \in C_k} S_{ij} =& 2 \sum_{k=1}^{K} \sum_{j \in C_k} w_{ik} [I_{\gamma_{ik}=0} + I_{\gamma_{ik}=1} I_{x_{ij} > \mu_i + \frac{\delta_{ik}}{2}} + I_{\gamma_{ik}=1} I_{x_{ij} \leq \mu_i + \frac{\delta_{ik}}{2}}] \\ =& 2 \sum_{k=1}^{K} \sum_{j \in C_k} w_{ik} > 0,\end{aligned}$$

this $\hat{\mu}_i$ is a local minimum point. Again, we will use this $\hat{\mu}_i$ for our iteration and ignore those non-differentiable points.

We do not worry too much about the local instead of global minimum solution at each updating step because of two reasons. First, even if the global minimum point is identified and used at each updating step, due to the non-convexity of the optimization problem that scSorter tries to solve, the solution resulting from the whole iterative algorithm is still a local minimum, anyway. Second, the current formulas ($\hat{\delta}_{ik}$ and $\hat{\mu}_i$) seem to be highly interpretable: δ is updated by the mean of the differences between the observed expression and the baseline expression in the cells with elevated expression, and μ is the mean expression level of all cells, with the elevated component of expression removed.

The first alternative approach for the optimization problem

The first approach finds $\boldsymbol{C} = \{C_k\}_{k=1,\dots,K}$ and $\boldsymbol{\mu} = \{\mu_{ik}\}_{i=1,\dots,g+h,k=1,\dots,K}$ that

$$\text{minimize} \quad \sum_{i=1}^{g}\sum_{k=1}^{K}\sum_{j\in C_k} w_{ik}(x_{ij}-\mu_{ik})^2 + \sum_{i=g+1}^{g+h}\sum_{k=1}^{K}\sum_{j\in C_k}(x_{ij}-\mu_{ik})^2 \tag{9}$$

$$\text{subject to} \quad \left(\mu_{ik} - \frac{1}{N}\sum_{j=1}^{N} x_{ij}\right) I_{\gamma_{ik}=1} \geq 0, \qquad i = 1,\dots,g; k = 1,\dots,K. \tag{10}$$

w_{ik} is a pre-specified weight (a positive constant) for marker gene i of known cell type k, $i = 1,\dots,g$. γ_{ik} is an indicator with $\gamma_{ik} = 1$ denoting that gene i is a marker gene of cell type k and $\gamma_{ik} = 0$ otherwise.

The following algorithm that alternatively updates the cluster assignment and the cluster means is used:

1. Keeping $\boldsymbol{\mu}$ unchanged, update $\boldsymbol{C}$. This can be done by assigning cell j to the cluster that gives the smallest cost:

$$\sum_{i=1}^{g} w_{ik}(x_{ij}-\mu_{ik})^2 + \sum_{i=g+1}^{g+h}(x_{ij}-\mu_{ik})^2.$$

2. Keeping $\boldsymbol{C}$ unchanged, update $\boldsymbol{\mu}$. Note that our cost function is separable on i (i.e. gene), and thus solving $\boldsymbol{\mu}$ can be done by solving it for each gene independently.

   For marker genes, the Lagrangian for gene i is defined as:

$$L(\mu_{ik}, \lambda) = \sum_{k=1}^{K} \sum_{j \in C_k} w_{ik}(x_{ij} - \mu_{ik})^2 - \sum_{k=1}^{K} \lambda_{ik} \left(\mu_{ik} - \frac{1}{N} \sum_{j=1}^{N} x_{ij} \right) I_{\gamma_{ik}=1}. \tag{11}$$

   The KKT conditions are as follows:

   (a) $\frac{\partial}{\partial \mu_{ik}} L(\mu_{ik}, \lambda) = -\sum_{j \in C_k} 2w_{ik}(x_{ij} - \mu_{ik}) - \lambda_{ik} I_{\gamma_{ik}=1} = 0.$

   (b) $\lambda_{ik} I_{\gamma_{ik}=1} \geq 0.$

   (c) $-\lambda_{ik} \left(\mu_{ik} - \frac{1}{N} \sum_{j=1}^{N} x_{ij} \right) I_{\gamma_{ik}=1} = 0.$

   (d) $\left(\mu_{ik} - \frac{1}{N} \sum_{j=1}^{N} x_{ij} \right) I_{\gamma_{ik}=1} \geq 0.$

   We start from examining condition (b). First consider the case $\lambda_{ik} I_{\gamma_{ik}=1} = 0$, which leads to the solution

$$\hat{\mu}_{ik} = \frac{1}{n_k} \sum_{j \in C_k} x_{ij}. \tag{12}$$

   This $\hat{\mu}_{ik}$ is the final solution if condition (d) is satisfied. Otherwise, $\frac{1}{n_k} \sum_{j \in C_k} x_{ij} < \frac{1}{N} \sum_{j=1}^{N} x_{ij}$, which means we need to consider the other case of condition (b), $\lambda_{ik} I_{\gamma_{ik}=1} > 0$. Combined with condition (c), the solution is now:

$$\hat{\mu}_{ik} = \frac{1}{N} \sum_{j=1}^{N} x_{ij}. \tag{13}$$

   Further checking λ_{ik} gives

$$\lambda_{ik} = -2w_{ik} n_k \left(\frac{1}{n_k} \sum_{j \in C_k} x_{ij} - \frac{1}{N} \sum_{j=1}^{N} x_{ij} \right) > 0, \tag{14}$$

   which meets the condition (b) and implies that $\hat{\mu}_{ik}$ is a valid solution.

The solution for μ_{ik} based on the above discussion could be summarized as

$$\hat{\mu}_{ik} = max\left(\frac{1}{n_k}\sum_{j\in C_k} x_{ij}, \frac{1}{N}\sum_{j=1}^{N} x_{ij}\right). \tag{15}$$

For non-marker genes, μ_{ik} has a simple closed-form solution:

$$\frac{\partial}{\partial \mu_{ik}}\sum_{j\in C_k}(x_{ij}-\mu_{ik})^2 = -2\sum_{j\in C_k}(x_{ij}-\mu_{ik}) = 0, \tag{16}$$

which results in

$$\hat{\mu}_{ik} = \frac{1}{n_k}\sum_{j\in C_k} x_{ij}. \tag{17}$$

The second alternative approach for the optimization problem

The second approach finds $\boldsymbol{C} = \{C_k\}_{k=1,\ldots,K}$ and $\boldsymbol{\mu} = \{\mu_{ik}\}_{i=1,\ldots,g+h,k=1,\ldots,K}$ that

$$\text{minimize} \quad \sum_{i=1}^{g}\sum_{k=1}^{K}\sum_{j\in C_k} w_{ik}(x_{ij}-\mu_{ik})^2 + \sum_{i=g+1}^{g+h}\sum_{k=1}^{K}\sum_{j\in C_k}(x_{ij}-\mu_{ik})^2 \tag{18}$$

$$\text{subject to} \quad \min_{k\in\{k;I_{\gamma_{ik}=1}\}} \mu_{ik} \geq \max_{k\in\{k;I_{\gamma_{ik}=0}\}} \mu_{ik}, \qquad i = 1,\ldots,g. \tag{19}$$

w_{ik} is a pre-specified weight (a positive constant) for marker gene i of known cell type k, $i = 1,\ldots,g$. γ_{ik} is an indicator with $\gamma_{ik} = 1$ denoting that gene i is a marker gene of cell type k and $\gamma_{ik} = 0$ otherwise.

The following algorithm that alternatively updates the cluster assignment and the cluster means is used:

1. Keeping $\boldsymbol{\mu}$ unchanged, update $\boldsymbol{C}$. This can be done by assigning cell j to the cluster

that gives the smallest cost:

$$\sum_{i=1}^{g} w_{ik}(x_{ij} - \mu_{ik})^2 + \sum_{i=g+1}^{g+h} (x_{ij} - \mu_{ik})^2.$$

2. Keeping $\boldsymbol{C}$ unchanged, update $\boldsymbol{\mu}$. Note that our cost function is separable on i (i.e. gene), and thus solving $\boldsymbol{\mu}$ can be done by solving it for each gene independently.

   For marker genes, the Lagrangian for gene i is defined as:

$$L(\mu_{ik}, \lambda) = \sum_{k=1}^{K} \sum_{j \in C_k} w_{ik}(x_{ij} - \mu_{ik})^2 - \sum_{k=1}^{K} \sum_{k'=1}^{K} \lambda_{ikk'}(\mu_{ik} - \mu_{ik'}) I_{\gamma_{ik}=1} I_{\gamma_{ik'}=0}. \quad (20)$$

   The KKT conditions are as follows:

   (a) $\frac{\partial}{\partial \mu_{ik}} L(\mu_{ik}, \lambda) = -\sum_{j \in C_k} 2w_{ik}(x_{ij} - \mu_{ik}) - \sum_{k' \in \{k; I_{\gamma_{ik}=0}\}} \lambda_{ikk'} = 0$ for $k \in \{k; I_{\gamma_{ik}=1}\}$.

   (b) $\frac{\partial}{\partial \mu_{ik'}} L(\mu_{ik'}, \lambda) = -\sum_{j \in C_{k'}} 2w_{ik'}(x_{ij} - \mu_{ik'}) + \sum_{k \in \{k; I_{\gamma_{ik}=1}\}} \lambda_{ikk'} = 0$ for $k' \in \{k; I_{\gamma_{ik}=0}\}$.

   (c) $\lambda_{ikk'} \geq 0$ for $k \in \{k; I_{\gamma_{ik}=1}\}, k' \in \{k; I_{\gamma_{ik}=0}\}$.

   (d) $-\lambda_{ikk'}(\mu_{ik} - \mu_{ik'}) = 0$ for $k \in \{k; I_{\gamma_{ik}=1}\}, k' \in \{k; I_{\gamma_{ik}=0}\}$.

   (e) $\mu_{ik} - \mu_{ik'} \geq 0$ for $k \in \{k; I_{\gamma_{ik}=1}\}, k' \in \{k; I_{\gamma_{ik}=0}\}$.

   Here, we introduce k and k' to make it easy to distinguish between clusters where gene i is considered as a marker gene and clusters where gene i is not considered as a marker gene.

   We start from examining condition (c). First consider the case when all $\lambda_{ikk'} = 0$, which leads to

$$\hat{\mu}_{ik} = \frac{1}{n_k} \sum_{j \in C_k} x_{ij}. \quad (21)$$

   This $\hat{\mu}_{ik}$ is the final solution if condition (e) is satisfied. Otherwise, $\hat{\mu}_{ik} - \hat{\mu}_{ik'} < 0$ holds true for at least one pair of k, k'. In this case, we should consider the counterpart case

of condition (c) $\lambda_{ikk'} > 0$, which implies $\mu_{ik} - \mu_{ik'} = 0$ with condition (d) taking into account. That is to say, the cluster of k and $k^{'}$ share the same cluster center estimate for gene i. In other words, if $\lambda_{ikk'} > 0$ is considered for a pair of clusters, these clusters will share center estimate.

The solution of this optimization problem follows this idea by calculating initial estimates of μ_{ik} assuming $\hat{\mu}_{ik} - \hat{\mu}_{ik'} \geq 0$ holds true for all pairs of k, $k^{'}$. Then, condition (e) is examined for each pair of k, $k^{'}$. The initial estimates of μ_{ik} that violate condition (e) are updated by considering the counterpart case $\lambda_{ikk'} > 0$ of condition (c).

First consider the simplest case in which $\hat{\mu}_{ik} - \hat{\mu}_{ik'} < 0$ occurs for one and only one pair of k, $k^{'}$. For all clusters other than k and $k^{'}$, the solution is

$$\hat{\mu}_{ik} = \frac{1}{n_k} \sum_{j \in C_k} x_{ij}. \tag{22}$$

For clusters k and $k^{'}$, we need to consider the case $\lambda_{ikk'} > 0$ of condition (c) and have

$$-\sum_{j \in C_k} 2w_{ik}(x_{ij} - \mu_{ik}) - \lambda_{ikk'} = 0$$

and

$$-\sum_{j \in C_{k'}} 2w_{ik'}(x_{ij} - \mu_{ik'}) + \lambda_{ikk'} = 0,$$

which leads to the solution

$$\hat{\mu}_{ikk'} = \hat{\mu}_{ik} = \hat{\mu}_{ik'} = \frac{\sum_{j \in C_k} w_{ik} x_{ij} + \sum_{j \in C_{k'}} w_{ik'} x_{ij}}{\sum_{j \in C_k} w_{ik} + \sum_{j \in C_{k'}} w_{ik'}}.$$

Note that $\lambda_{ikk'} > 0$ implies $\hat{\mu}_{ik} = \hat{\mu}_{ik'}$ as a result of condition (d).

For general cases when multiple $\hat{\mu}_{ik} - \hat{\mu}_{ik'} < 0$ holds true, the idea is similar. Those

clusters will share the same cluster center estimate as the case $\lambda_{ikk'} > 0$ of condition (c) will be considered for those clusters which implies $\hat{\mu}_{ik} - \hat{\mu}_{ik'} = 0$. To solve this optimization problem, the key is to figure out clusters that share center estimate, which could be achieved by first calculating $\hat{\mu}_{ik}$ assuming all $\lambda_{ikk'} = 0$. Then, order all $\hat{\mu}_{ik}$ in descending order which guarantees that $\hat{\mu}_{i(k)} > \hat{\mu}_{i(k+1)}$ for $k \in \{k; I_{\gamma_{ik}=1}\}$ and $\hat{\mu}_{i(k')} > \hat{\mu}_{i(k'+1)}$ for $k^{'} \in \{k; I_{\gamma_{ik}=0}\}$. Denote the size of $\{k; I_{\gamma_{ik}=0}\}$ as K_i^0 and the size of $\{k; I_{\gamma_{ik}=1}\}$ as K_i^1. The following three statements hold true:

(a) If $\lambda_{i(k)(k')} > 0$ should be considered, then $\lambda_{i(k+1)(k')} > 0, \lambda_{i(k+2)(k')} > 0, ..., \lambda_{i(K_i^1)(k')} > 0$ should also be considered.

(b) If $\lambda_{i(k)(k')} > 0$ should be considered, then $\lambda_{i(k)(k'-1)} > 0, \lambda_{i(k)(k'-2)} > 0, ..., \lambda_{i(k)(1')} > 0$ should also be considered.

(c) For $k_0 < k$, if $\lambda_{i(k_0)(1')} > 0$ and $\lambda_{i(k)(k')} > 0$ should be considered, then $\lambda_{i(k_0)(k')} > 0$ should also be considered. The reason for this statement is that if $\lambda_{i(k)(k')} > 0$ should be considered, then $\lambda_{i(k)(1')} > 0$ should be considered following statement (b), which implies $\hat{\mu}_{i(k_0)} - \hat{\mu}_{i(1')} = 0$, $\hat{\mu}_{i(k)} - \hat{\mu}_{i(k')} = 0$, and $\hat{\mu}_{i(k)} - \hat{\mu}_{i(1')} = 0$ following condition (d). Therefore, $\hat{\mu}_{i(k_0)} - \hat{\mu}_{i(k')} = 0$ holds true and $\lambda_{i(k_0))(k')} > 0$ should be considered.

With above statements detecting all clusters that share center estimate, the complete solution of this problem is as follows: First calculate μ_{ik} assuming all $\lambda_{ikk'} = 0$. If for every $k \in \{k; I_{\gamma_{ik}=1}\}$ and $k^{'} \in \{k; I_{\gamma_{ik}=0}\}$, $\hat{\mu}_{ik} \geq \hat{\mu}_{ik'}$, the solution is found. If there are cases that $\hat{\mu}_{ik} < \hat{\mu}_{ik'}$, sort the $\hat{\mu}_{ik}$'s and $\hat{\mu}_{ik'}$'s in descending order which results in $\hat{\mu}_{i(k)}$'s and $\hat{\mu}_{i(k')}$'s. Let $\hat{\mu}_{i(k_0)}$ be the largest marker genes' cluster center that is smaller than $\hat{\mu}_{i(k')}$ for some $k^{'} \in \{k; I_{\gamma_{ik}=0}\}$. In other words, no $\hat{\mu}_{i(k)} < \hat{\mu}_{i(k')}$ occurs for $k = k_0 - 1, ..., 1$. Similarly, let $\hat{\mu}_{i(k_0^{'})}$ be the smallest non-marker genes' cluster center that is larger than $\hat{\mu}_{i(k)}$ for some $k \in \{k; I_{\gamma_{ik}=1}\}$. In other words, no $\hat{\mu}_{i(k)} < \hat{\mu}_{i(k')}$ occurs for $k^{'} = k_0^{'} + 1, ..., K_i^0$. The solution of the problem is then as follows:

For $k \in \{k; I_{\gamma_{ik}=1}\}$ and $k < k_0$,

$$\hat{\mu}_{i(k)} = \frac{1}{n_{(k)}} \sum_{j \in C_{(k)}} x_{ij}. \tag{23}$$

For $k^{'} \in \{k; I_{\gamma_{ik}=0}\}$ and $k^{'} > k_0^{'}$,

$$\hat{\mu}_{i(k')} = \frac{1}{n_{(k')}} \sum_{j \in C_{(k')}} x_{ij}. \tag{24}$$

For $k \in \{k; I_{\gamma_{ik}=1}\}$ and $k \geq k_0$ and $k^{'} \in \{k; I_{\gamma_{ik}=0}\}$ and $k^{'} \leq k_0^{'}$, the above discussion ensures clusters within this range all share the same center estimate:

$$\hat{\mu}_{i(k)} = \hat{\mu}_{i(k')} = \frac{\sum_{j \in C_{(k_0)}} w_{ik} x_{ij} + \ldots + \sum_{j \in C_{(K_i^1)}} w_{ik} x_{ij} + \sum_{j \in C_{(1')}} w_{ik} x_{ij} + \ldots + \sum_{j \in C_{(k_0')}} w_{ik} x_{ij}}{\sum_{j \in C_{(k_0)}} w_{ik} + \ldots + \sum_{j \in C_{(K_i^1)}} w_{ik} + \sum_{j \in C_{(1')}} w_{ik} + \ldots + \sum_{j \in C_{(k_0')}} w_{ik}}. \tag{25}$$

For non-marker genes, μ_{ik} has a simple closed-form solution:

$$\frac{\partial}{\partial \mu_{ik}} \sum_{j \in C_k} (x_{ij} - \mu_{ik})^2 = -2 \sum_{j \in C_k} (x_{ij} - \mu_{ik}) = 0, \tag{26}$$

which results in

$$\hat{\mu}_{ik} = \frac{1}{n_k} \sum_{j \in C_k} x_{ij}. \tag{27}$$

The third alternative approach for the optimization problem

The third approach finds $\boldsymbol{C} = \{C_k\}_{k=1,\ldots,K}$, $\boldsymbol{\mu} = \{\mu_i\}_{i=1,\ldots,g} \cup \{\mu_{ik}\}_{i=g+1,\ldots,g+h,k=1,\ldots,K}$, and $\boldsymbol{\delta} = \{\delta_{ik}\}_{i=1,\ldots,g;k=1,\ldots,K}$ that

$$\text{minimize} \quad \sum_{i=1}^{g}\sum_{k=1}^{K}\sum_{j\in C_k} w_{ik}\left[(x_{ij}-\mu_i)^2 I_{\gamma_{ik}=0} + (x_{ij}-\mu_i-\delta_{ik})^2 I_{\gamma_{ik}=1}\right] \tag{28}$$

$$+\sum_{i=g+1}^{g+h}\sum_{k=1}^{K}\sum_{j\in C_k}(x_{ij}-\mu_{ik})^2 \tag{29}$$

$$\text{subject to} \quad \delta_{ik} \geq 0, \qquad i=1,\ldots,g; k=1,\ldots,K. \tag{30}$$

w_{ik} is a pre-specified weight (a positive constant) for marker gene i of known cell type k, $i = 1, \ldots, g$. γ_{ik} is an indicator with $\gamma_{ik} = 1$ denoting that gene i is a marker gene of cell type k and $\gamma_{ik} = 0$ otherwise.

The following algorithm that alternatively updates the cluster assignment and the cluster means is used:

1. Keeping $\boldsymbol{\mu}$ and $\boldsymbol{\delta}$ unchanged, update $\boldsymbol{C}$. This can be done by assigning cell j to the cluster that gives the smallest cost:

$$\sum_{i=1}^{g} w_{ik}\left[(x_{ij}-\mu_i)^2 I_{\gamma_{ik}=0} + (x_{ij}-\mu_i-\delta_{ik})^2 I_{\gamma_{ik}=1}\right] + \sum_{i=g+1}^{g+h}(x_{ij}-\mu_{ik})^2.$$

2. Keeping $\boldsymbol{C}$ unchanged, update $\boldsymbol{\mu}$ and $\boldsymbol{\delta}$. Note that our cost function is separable on i (i.e. gene), and thus solving $\boldsymbol{\mu}$ and $\boldsymbol{\delta}$ can be done by solving them for each gene independently.

   For marker genes, the Lagrangian for gene i is defined as:

$$L(\mu_i,\delta_{ik},\lambda) = \sum_{k=1}^{K}\sum_{j\in C_k} w_{ik}\left[(x_{ij}-\mu_i)^2 I_{\gamma_{ik}=0} + (x_{ij}-\mu_i-\delta_{ik})^2 I_{\gamma_{ik}=1}\right] - \sum_{k=1}^{K}\lambda_{ik}\delta_{ik}I_{\gamma_{ik}=1}. \tag{31}$$

The KKT conditions are as follows:

(a) $\frac{\partial}{\partial \mu_i} L(\mu_i, \delta_{ik}, \lambda) =$
$-2\sum_{k=1}^{K}\sum_{j\in C_k} w_{ik}\left[(x_{ij}-\mu_i)I_{\gamma_{ik}=0} + (x_{ij}-\mu_i-\delta_{ik})I_{\gamma_{ik}=1}\right] = 0.$

(b) $\frac{\partial}{\partial \delta_{ik}} L(\mu_i, \delta_{ik}, \lambda) = -2\sum_{j\in C_k} w_{ik}(x_{ij}-\mu_i-\delta_{ik})I_{\gamma_{ik}=1} - \lambda_{ik}I_{\gamma_{ik}=1} = 0.$

(c) $\lambda_{ik}I_{\gamma_{ik}=1} \geq 0.$

(d) $-\lambda_{ik}\delta_{ik}I_{\gamma_{ik}=1} = 0.$

(e) $\delta_{ik}I_{\gamma_{ik}=1} \geq 0.$

We start from examining condition (c). First consider the case $\lambda_{ik}I_{\gamma_{ik}=1} = 0$, which lead to the solution

$$\hat{\delta}_{ik} = \frac{1}{n_k}\sum_{j\in C_k} x_{ij} - \hat{\mu}_i, \tag{32}$$

where

$$\hat{\mu}_i = \frac{\sum_{j=1}^{N} w_{ik}x_{ij} - \sum_{k=1}^{K}\sum_{j\in C_k} w_{ik}\hat{\delta}_{ik}I_{\gamma_{ik}=1}}{\sum_{k=1}^{K}\sum_{j\in C_k} w_{ik}}. \tag{33}$$

The $\hat{\mu}_i$ and $\hat{\delta}_{ik}$ are the final solution if condition (e) is satisfied. Otherwise, $\hat{\delta}_{ik} = \frac{1}{n_k}\sum_{j\in C_k} x_{ij} - \hat{\mu}_i < 0$ holds true for some clusters k , and the corresponding case $\lambda_{ik}I_{\gamma_{ik}=1} > 0$ of condition (c) should be considered, which implies $\delta_{ik} = 0$ together with condition (d). If $\delta_{ik} = 0$ and $\gamma_{ik} = 1$, $\lambda_{ik} = -2n_k w_{ik}\left(\frac{1}{n_k}\sum_{j\in C_k} x_{ij} - \hat{\mu}_i\right) > 0$ which meets condition (c) and implies that $\hat{\delta}_{ik} = 0$ is a valid solution in this case. Therefore, the final solution is:

$$\hat{\delta}_{ik} = \max\left(\frac{1}{n_k}\sum_{j\in C_k} x_{ij} - \hat{\mu}_i, 0\right), \tag{34}$$

where

$$\hat{\mu}_i = \frac{\sum_{j=1}^{N} w_{ik}x_{ij} - \sum_{k=1}^{K}\sum_{j\in C_k} w_{ik}\hat{\delta}_{ik}I_{\gamma_{ik}=1}}{\sum_{k=1}^{K}\sum_{j\in C_k} w_{ik}}. \tag{35}$$

The above equations show that μ_i and δ_{ik} do not have a closed-form solution, and we

solve them by repetitively updating one given the other using equation 34 and 35 until convergence.

For non-marker genes, μ_{ik} has a simple closed-form solution:

$$\frac{\partial}{\partial \mu_{ik}} \sum_{j \in C_k} (x_{ij} - \mu_{ik})^2 = -2 \sum_{j \in C_k} (x_{ij} - \mu_{ik}) = 0, \tag{36}$$

which results in

$$\hat{\mu}_{ik} = \frac{1}{n_k} \sum_{j \in C_k} x_{ij}. \tag{37}$$

Details on simulated data

The simulated datasets used in this study were generated by first using Splatter and then modifying the resulting datasets to mimic the excessive zeros on marker genes observed in real datasets. In the first step, we used the version of Splatter modified by the author of CellAssign (`https://github.com/Irrationone/splatter`) to deal with the extreme log fold change they observed in real data. As in CellAssign, the majority of Splatter parameters were estimated by applying Splatter function splatEstimate() on 10x Genomics dataset "4k Pan T Cells from a Healthy Donor". The parameters controlling the location and the scale of differential-expression genes followed the estimates from the CellAssign paper that were based on naïve CD4+ and naïve CD8+ T cells of the 68k PBMC dataset. The parameter for the proportion of genes differentially expressed between cell types was set as 0.5. Among the top ten genes with the highest fold changes of one cell type compared to other cell types, two to five were randomly selected as marker genes of that cell type.

One issue we observed on data generated by Splatter was that the marker genes have much less numbers of zeros than in the real PBMC dataset. The left panel of Figure S9 shows the percentage of zeros observed on marker genes in the cell type where these genes are marker genes (x axis) and the percentage of zeros observed on marker genes in the cell

types where these genes are not marker genes (y axis), in both the PBMC dataset ("Real Data", red) and a simulated dataset using Splatter ("Simulation", cyan). (Notice that the numbers of red points and cyan points in the plot are generally not the same, since the number of marker genes in simulated dataset is a random number and thus does not need to equal the number of marker genes in the real data.) It is clear that the percentage of zeros in marker genes, especially in the cell types where they are not marker genes, is severely lower in the simulated data (compared to real data). To correct this inconsistency, two logistic curves were fitted using the mean of non-zero expression of marker genes in the cell type where they are marker genes to describe the percentage of zeros on marker genes, in the cell type where they are marker genes and in the cell types where they are not marker genes, respectively. Then these curves were used to give the expected percentages of zeros in the simulated dataset. If the percentage of zeros of a selected marker gene from a dataset generated by Splatter was too low, non-zero values were randomly selected and set to zero. After this correction, the resulting dataset resembles the real dataset much better in the sense of percentages of zeros on marker genes (right panel of Figure S9).

Cells from each cell type were generated using the way described above. To determine the number of cells in each cell type, $k-1$ random numbers were generated from Uniform distribution $U(0,1)$ and sorted from the smallest to largest. Let them be $p_{(1)}$, $p_{(2)}$, $\ldots$, and $p_{(k-1)}$. Then the proportion of cells in the K cell types are set as $p_{(1)}$, $p_{(2)} - p_{(1)}$, $\ldots$, $p_{(k-1)} - p_{(k-2)}$, and $1 - p_{(k-1)}$.

Under each simulation scenario, 50 datasets were generated with $I = 10,000$ genes and $N = 5,000$ cells from $K = 10$ cell types following the above approach. For scenario 2, the expression data of one to three cell types were removed from the generated dataset to simulate the case when redundant prior cell type information was provided. For scenario 3, the identity of marker genes of one to three cell types was deleted to simulate the case when unknown cell types existed.

Details on software usage

SCINA (version 1.2.0) was applied to all simulated data and real datasets with parameter rm_overlap = 0 and the other parameters following the default settings.

Garnett (version 0.1.16) was applied following the recommended procedure from its vignette. check_markers() function was first applied to screen the marker genes. If a marker gene was labeled as ’High overlap’ and had a low “percent of assignment” level (colored dark blue in the diagnostic plot), it was not used by Garnett. Garnett was then applied with the filtered list of marker genes with parameters following the default settings except for parameter num_unknown that determined the number of cells selected as candidates for the unknown cell type. The default value for num_unknown was 500, which was too large for the dataset with a small sample size. Therefore, it was chosen with dataset sample size under consideration. For all simulated data, num_unknown = 50. For Rosenberg dataset, num_unknown = 200. For TM pancreas dataset, num_unknown = 25. For MCA lung dataset, num_unknown = 50. For PBMC dataset, num_unknown = 500. For CBMC dataset, num_unknown = 50.

CellAssign (version 0.99.21) was applied by first generating a SingleCellExperiment object that contained the whole expression matrix. The required size factor was calculated by the computeSumFactors() function from scran package (version 1.14.6). Design matrix was generated explicitly with the marker_list_to_mat() function from CellAssign package. Cell type assignment was then conducted by the cellassign() function with learning rate set to 0.01. The rest parameters followed their defaults. For Rosenberg, PBMC, CBMC, the parameter num_runs (that determines the number of EM optimizations to perform) was increased to 20 so that a good local optimum could be reached.

SC3 (version 1.14.0) was applied by first generating a SingleCellExperiment object that contained the whole expression matrix. The data was then normalized by the logNormCounts() function from scater package (version 1.14.0). Clustering was conducted by the

main function sc3() with the number of clusters estimated by the sc3_estimate_k() function from SC3 package. To map each cluster resulted from SC3 to predefined cell types, the Pearson correlation coefficient between mean expression of marker genes of each cluster and the indicator vector of marker genes of each predefined cell type was calculated. Each cluster was assigned to the cell type with the highest correlation coefficient. If none of the correlation coefficients between a cluster and predefined cell types was larger than 0.5, the cluster was assigned "Unknown". The combination of SC3 and this cell type assignment approach was used to analyze both simulated and real datasets. For PBMC dataset, SC3 had difficulty to process the full dataset (the armadillo module it depended on cannot handle the full size of this dataset and reported error message: *error: Mat::init(): requested size is too large*.) Therefore, a sample of size 30,000 were extracted from the dataset and were used as input for SC3. The result of SC3 on PBMC dataset was calculated from this subset of original dataset.

UMAP [1] figures in this paper were generated by RunUMAP() function of Seurat (version 3.1.0) with all parameters following the default settings. Real datasets were first normalized by NormalizedData() function of Seurat with all parameters following the default settings. RunPCA() function of Seurat was then applied on the normalized expression data. The first 10 principal components were used as input for RunUMAP().

Marker genes used for each real dataset

Rosenberg data: Oligo: Mbp, Opalin, Hapln2, Tmem2, Bmp4, Gpr17, Bcas1; OPC: Pdgfra, Mki67; Immune: Tgfbr1, Dock2, Mrc1, Ly86, Dab2, Cx3cr1; Vascular: Rgs5, Flt1, Kdr, Abcc9, Pdgfrb; VLMC: Pdgfra, Col1a1, Col1a2, Slc6a13, Slc47a1, Slc47a2, Sidt1, Adamtsl3; Astrocyte: Aldh1l1, Slc7a10, Prdm16, Gfap, Gria1; Ependyma: Dnah1, Dnah2, Dnah5, Dnah9, Dnah10, Dnah11, Foxj1; OEC: Mybpc1, Lama4, Col5a2, Runx1.

TM Pancreas data: Pancreatic A cells: Gcg, Mafb, Arx; Pancreatic B cells: Ins1, Ins2,

Slc2a2, Nkx6-1, Pdx1, Mafa; Pancreatic D cells: Pdx1, Sst, Hhex; Pancreatic PP cells: Ppy; Pancreatic Acinar cells: Amy2b, Cpa1, Ptf1a; Pancreatic Ductal cells: Krt19, Hnf1b, Spp1; Endothelial cells: Pecam1, Cdh5, Kdr; Immune: Ptprc; Pancreatic Stellate cells: Pdgfra, Pdgfrb.

MCA lung data: Alveolar: Sftpc, Sftpa1, Sftpb, Sfta2, Dram1, Ager, Igfbp2, Hopx, Clic5, Pdpn, Krt8, Emp2, Aqp5, Sftpd; B cells: Cd79a, Ms4a1, Cd79b, Ighd, Cd19, Jchain, Igha, Igkc, Ighm, Igkv2-109; Ciliated cells: Pclaf, Scgb1a1, Aldh1a1, Cyp2f2, Scgb3a1, Hp, Ccdc153, Tmem212, Foxj1, Ccdc17, Cdc20, Ube2c, Stmn1, Tubb5; Dendritic cells: Cd74, Cd68, Ms4a6c, Plac8, Bst2, Irf7, Irf5, Naaa, Irf8, Itgax, Itgae, Gngt2, Lst1, Itgb2, Cd209a, H2-eb1, Itgam, H2-aa, H2-ab1, Ccnb2, Fscn1, Ccl22, Nudt17, H2-m2, Syngr2; Endothelial: Eng, Kdr, Flt1, Cdh5, Pecam1, Car4, Vwf; Fibroblasts: Dcn, Col3a1, Fgf10, Tcf21, Hoxa5, Inmt, Gsn, Acta2, Myl9; Granulocytes: G0s2, Clec4d, S100a9, S100a8, Cd14, Ngp, Cd177, Ly6g, Ccl4, Ccl3, Il6, Cd69, Cd200r3; Macrophage: Pclaf, Cd74, Cd68, Ear2, Ear1, Marco, Siglecf, C1qc, C1qa, Pf4, Adgre1, Chil3, Ccna2, Elane, Mpo, Ctsg, Prtn3, Ms4a3; NK cells: Nkg7, Klra8, Klra4, Klrb1c, Klra13-ps; T cells: Trbc2, Cd8b1, Cd3d, Cd3g, Thy1, Cxcr6, Icos, S100a4, Il7r, Cdk1.

PBMC data: CD34: CD34, THY1, ENG, KIT, PROM1; NK cells: NCAM1, FCGR3A; Monocytes: CD14, FCGR1A, CD68, S100A12; B cells: CD19, MS4A1, CD79A; T cells: CD3D, CD3E, CD3G.

CBMC data: CD34: CD34, THY1, ENG, KIT, PROM1; NK cells: NCAM1, FCGR3A; Monocytes: CD14, FCGR1A, CD68, S100A12; B cells: CD19, MS4A1, CD79A; CD4 T cells: CD3D, CD3E, CD3G, CD4, FOXP3, IL2RA, IL7R, CCR10; CD8 T cells: CD3D, CD3E, CD3G, CD8A, CD8B, CRTAM.

A straightforward two-step approach (SC3+correlation)

In the first step, SC3 (version 1.14.0) was used to group cells into clusters in a completely unsupervised way. Note that the number of clusters was automatically determined by SC3 using its sc3_estimate_k() function.

In the second step, to map each cluster given by SC3 to predefined cell types, the Pearson correlation coefficient between the mean expression of marker genes of each cluster and the indicator vector of marker genes of each predefined cell type was calculated. Each cluster was assigned to the cell type with the highest correlation coefficient if this correlation coefficient was larger than 0.5. If none of the correlation coefficients between a cluster and predefined cell types was larger than 0.5, the cluster was assigned "Unknown".

Note that we used SC3 instead of Seurat for the first step. This is because the cell types of the majority of real datasets used in this study were determined by Seurat or a similar approach involved Louvain clustering, which would give Seurat unfair advantage on this comparison. Below we elaborate on this point.

In the majority of real datasets used in this study, the cell types and corresponding marker genes of these datasets are derived from clusters detected by Louvain algorithm, and therefore using Louvain algorithm or Seurat for the first step may suffer from data-snooping bias.

Here are some examples about how real datasets use Louvain clustering. Rosenberg data was processed with a Jaccard-Louvain algorithm [2], which computes k-nearest-neighbors for each cell based on principal components, then builds cell-cell similarity matrix with Jaccard index based on the number of shared neighbors between every cell, and finally conducts clustering using the Louvain algorithm. TM pancreas, MCA lung, and CBMC datasets were all analyzed following the recommended approach of Seurat, which also computes k-nearest-neighbor graph of each cell based on reduced principal components or projections from other dimension reduction methods, constructs a shared nearest neighbor graph

by calculating the neighborhood overlap with Jaccard index and conducts clustering with Louvain algorithm as last.

We thus chose SC3, which is a powerful and highly popular method for clustering single-cell RNA-seq data, and it is different enough from Louvain clustering.

The OPC and Oligo cell types in Rosenberg data

Figure S10 shows the heatmap of marker genes (of any of the known cell types) as well as highly variable genes, with the cells grouped according to the labels given by Garnett. Also given in the figure are the “true” cell types, that is, the cell types that were determined by the original paper that generated the dataset.

In the figure, the top nine marker genes are for the Oligo or the OPC cell types. We marked out *Mbp* (a marker gene for the Oligo cell type), *Bcas1* (a marker gene for the Oligo cell type), and *Pdgfra* (a marker gene for the OPC cell type), which are the only ones that have high expression in a significant proportions of cells. We see that overall the pre-assumed marker genes do not contain strong and consistent signals in differentiating the Oligo and OPC cell types. This is even more clearly the case for the cells in the rectangle: their expression levels on *Mbp* and *Pdgfra* are both consistently low, and only about one half of them have high expression on *Bcas1*.

However, the expression on the highly variable genes may give a more definite answer to the cell type assignment of the cells in the red rectangle. The expression profiles of these cells on the highly variable genes are highly similar to the OPC cells and very different from the Oligo cells. This strongly supports the assignment of these cells to the OPC cell type, and Garnett misclassified these cells. scSorter, in contrary, made the right assignment on these cells. Figure S11 shows the heatmap with the cells grouped according to the labels given by scSorter.

CellAssign may fail in highly imbalanced datasets

We were curious why CellAssign failed on PBMC data and Rosenberg data, and we believe we have figured out the reason: CellAssign seems to have difficulty in dealing with imbalanced datasets, that is, datasets that contains cell types of very different sizes (i.e., very different numbers of cells in different cell types). We illustrate this using PBMC data, which contains a cell type, T cells, that account for 67.97% of all cells. We subsampled these 64,341 T cells to smaller numbers so that the data was more balanced, and re-ran CellAssign. Table S3 gives the misclassification rates under subsamplings with different numbers of T cells. It is clear that the performance of CellAssign was acceptable when the number of T cells was comparable with other cell types, but as the data got more imbalanced, the performance of CellAssign deteriorated significantly.

References

[1] Leland McInnes, John Healy, and James Melville. Umap: Uniform manifold approximation and projection for dimension reduction. *arXiv preprint arXiv:1802.03426*, 2018.

[2] Karthik Shekhar, Sylvain W Lapan, Irene E Whitney, Nicholas M Tran, Evan Z Macosko, Monika Kowalczyk, Xian Adiconis, Joshua Z Levin, James Nemesh, Melissa Goldman, et al. Comprehensive classification of retinal bipolar neurons by single-cell transcriptomics. *Cell*, 166(5):1308–1323, 2016.

Tables

Dataset	Method		
	vst	mean-variance-plot	dispersion
Rosenberg	0.1746	0.1826	0.1646
TM pancreas	0.0550	0.0537	0.0537
MCA lung	0.0831	0.1000	0.0844
PBMC	0.0608	0.0597	0.0672
CBMC	0.1551	0.1629	0.1574

Table S1: Misclassification rate of scSorter under different methods for choosing highly variable genes on real datasets.

Dataset	Mean	Standard Deviation
Rosenberg	0.1928	0.0149
TM pancreas	0.0550	0.0000
MCA lung	0.0831	0.0002
PBMC	0.0608	0.0000
CBMC	0.1551	0.0000

Table S2: Mean and standard deviation of the misclassification rate of scSorter on real datasets, when scSorter was run ten times under different random seeds.

Number of T cells	9,686	19,686	29,686	39,686	49,686	59,686
Misclassification rate	0.2655	0.2220	0.2239	0.7570	0.7888	0.8129

Table S3: Misclassification rate of CellAssign on PBMC dataset with different levels of imbalance.

Figures

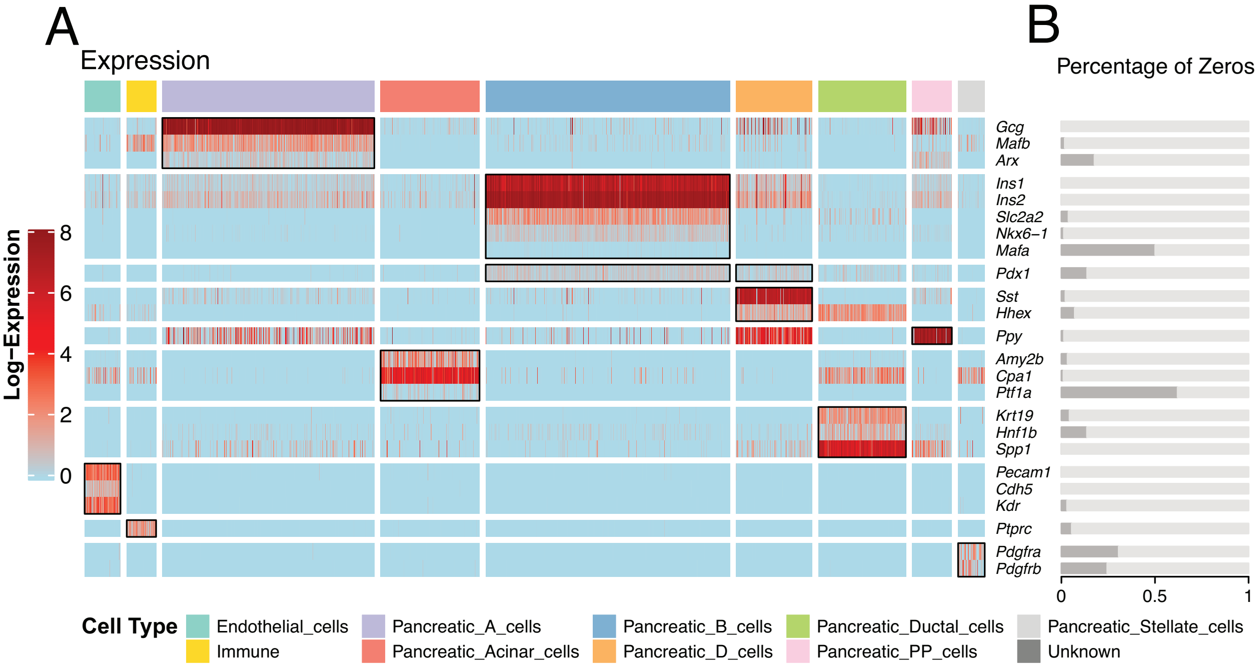

Figure S1: Visualization of TM pancreas data. A) Expression of cells from different cell types on marker genes. Columns are cells and rows are marker genes. Cells are grouped according to the (true) cell types that they belong to, with the colorful bar on the top of the expression matrix giving the cell type identities, and the marker genes are grouped according to the cell types that they correspond to. The white vertical and horizontal gaps separate cells from different types and marker genes corresponding to different cell types. The black boxes indicate the correspondences of cell types and marker genes. B) The bars show the percentages of cells that have zero expression in the corresponding cell type, for each marker gene.

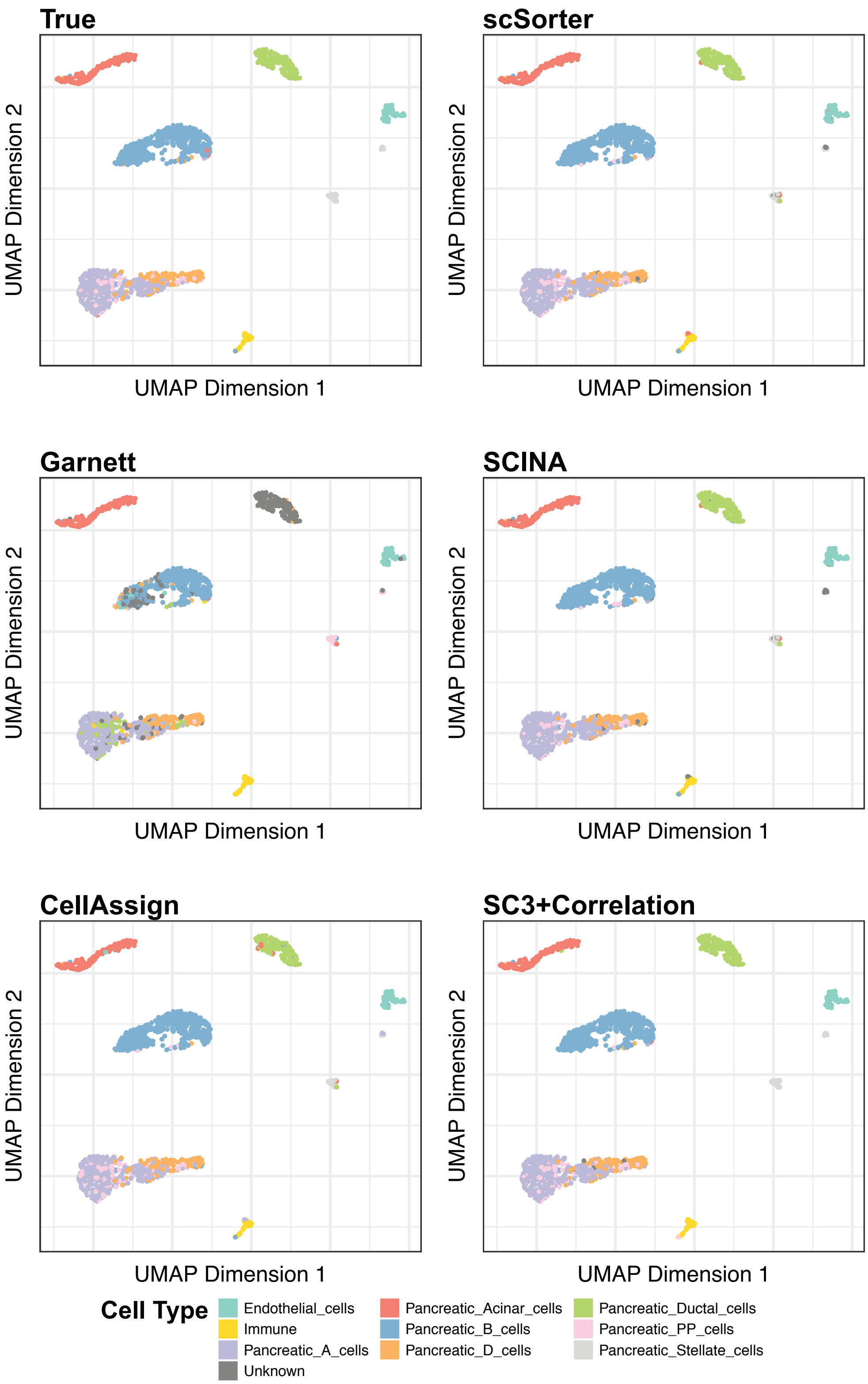

Figure S2: UMAP plots of TM pancreas dataset. The first panel is colored by true cell types. The rest five panels are colored by cell types assigned by the corresponding methods.

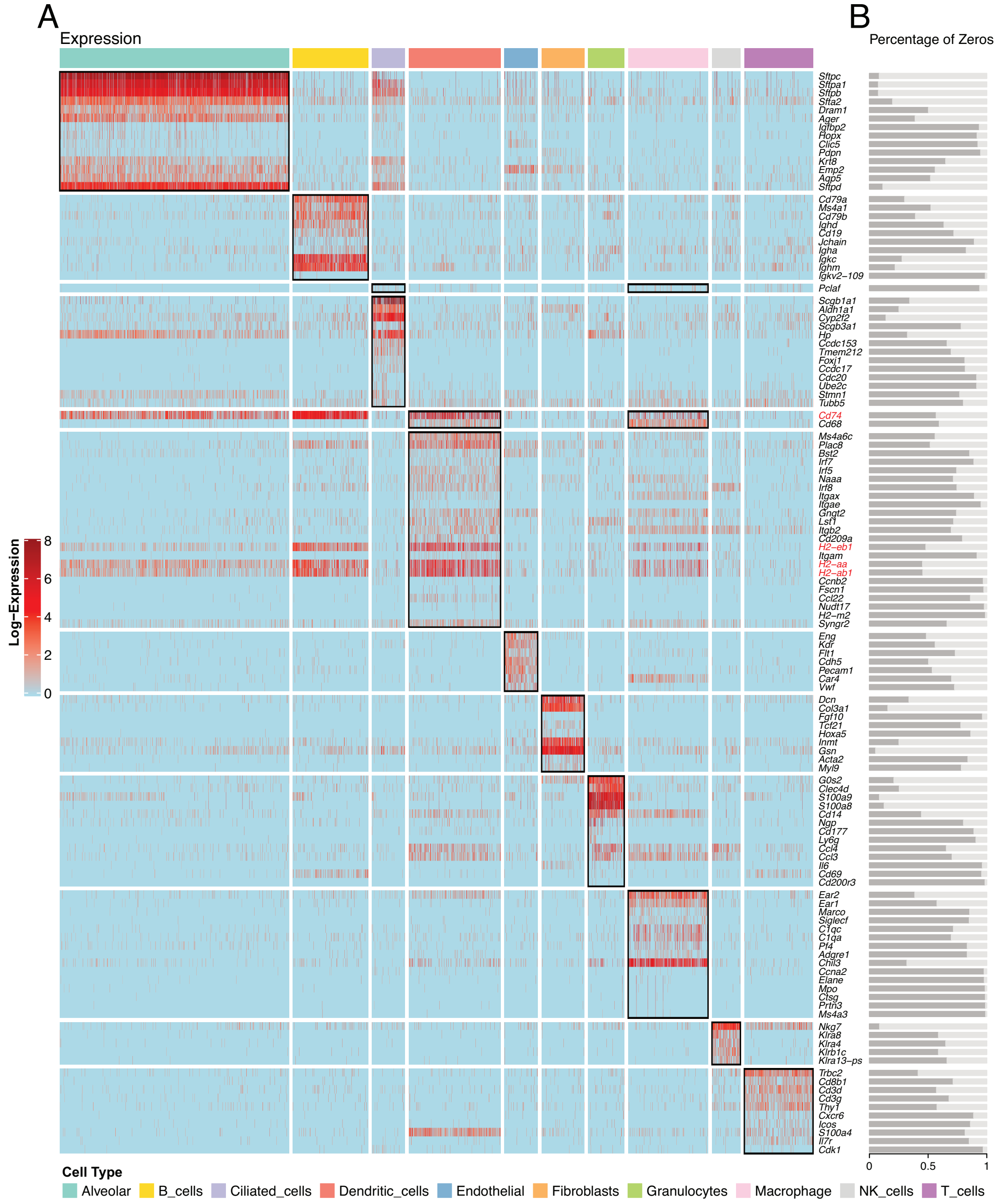

Figure S3: Visualization of MCA lung data. A) Expression of cells from different cell types on marker genes. Columns are cells and rows are marker genes. Cells are grouped according to the (true) cell types that they belong to, with the colorful bar on the top of the expression matrix giving the cell type identities, and the marker genes are grouped according to the cell types that they correspond to. The white vertical and horizontal gaps separate cells from different types and marker genes corresponding to different cell types. The black boxes indicate the correspondences of cell types and marker genes. B) The bars show the percentages of cells that have zero expression in the corresponding cell type, for each marker gene.

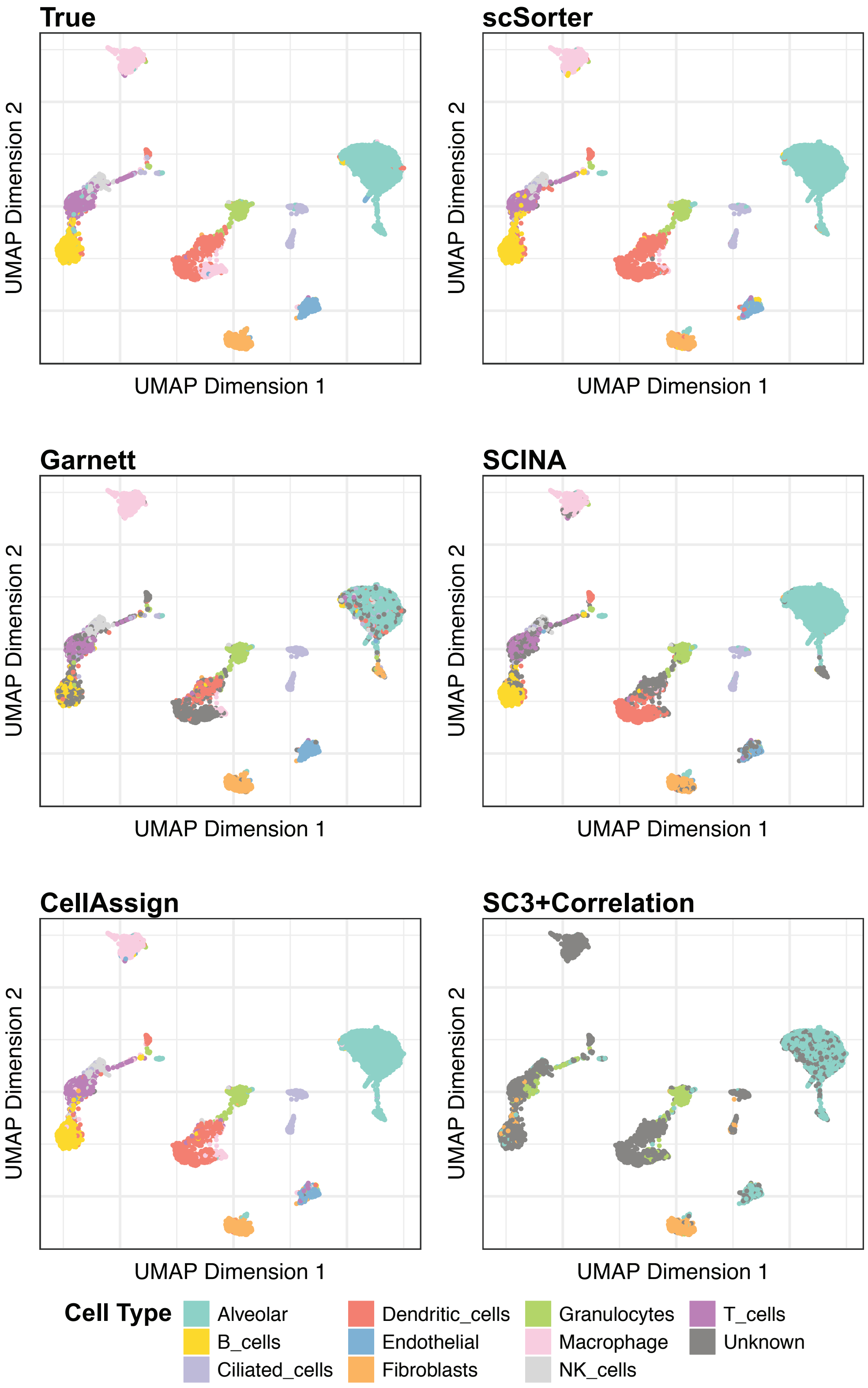

Figure S4: UMAP plots of MCA lung dataset. The first panel is colored by true cell types. The rest five panels are colored by cell types assigned by the corresponding methods.

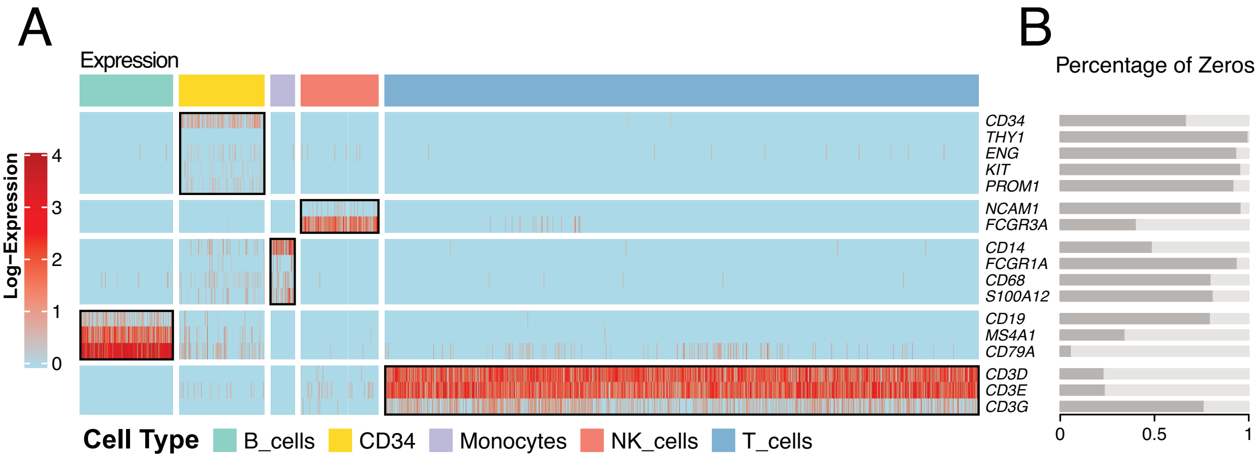

Figure S5: Visualization of PBMC data. A) Expression of cells from different cell types on marker genes. Columns are cells and rows are marker genes. Cells are grouped according to the (true) cell types that they belong to, with the colorful bar on the top of the expression matrix giving the cell type identities, and the marker genes are grouped according to the cell types that they correspond to. The white vertical and horizontal gaps separate cells from different types and marker genes corresponding to different cell types. The black boxes indicate the correspondences of cell types and marker genes. B) The bars show the percentages of cells that have zero expression in the corresponding cell type, for each marker gene.

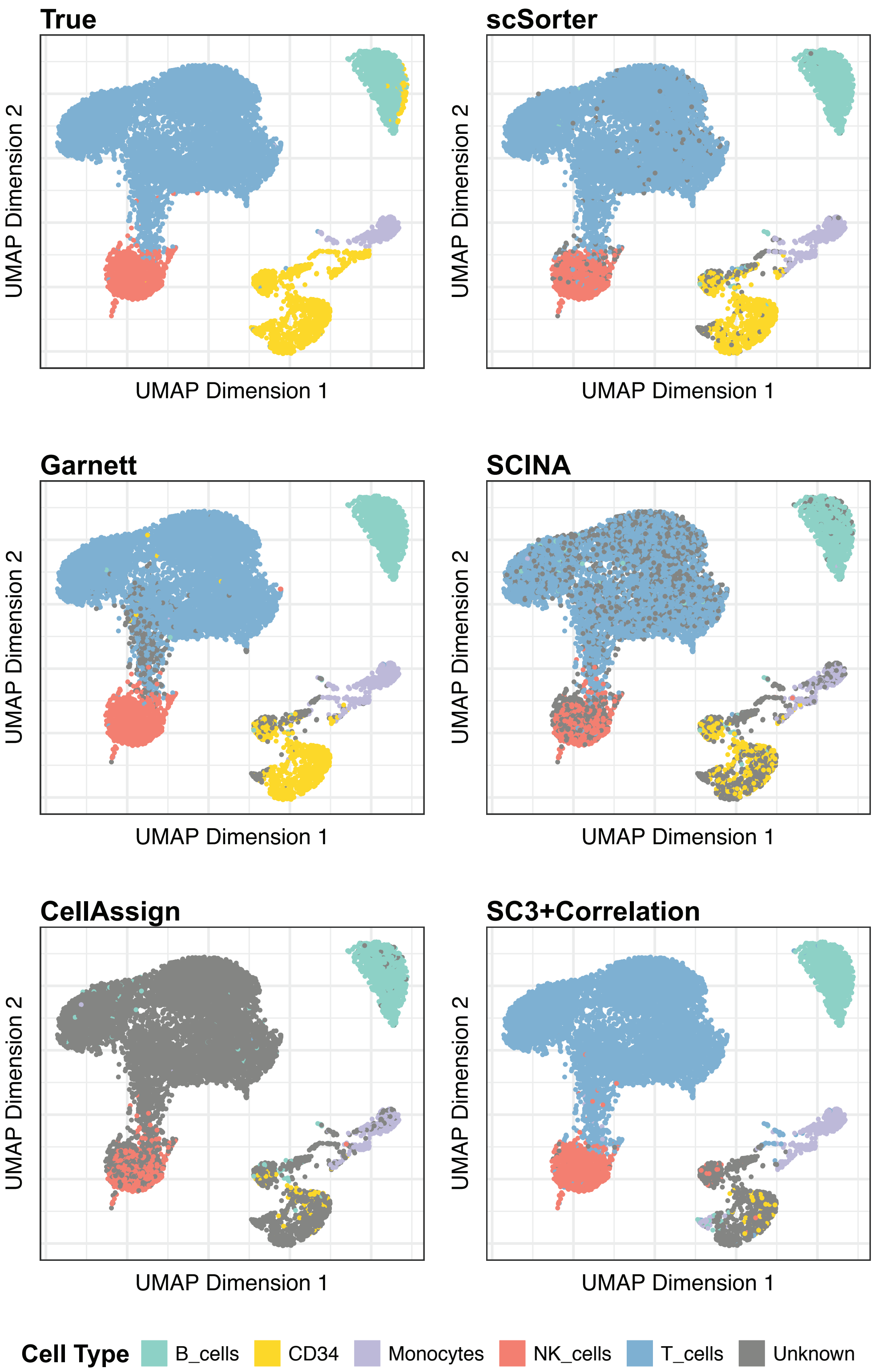

Figure S6: UMAP plots of PBMC dataset. The first panel is colored by true cell types. The rest five panels are colored by cell types assigned by the corresponding methods.

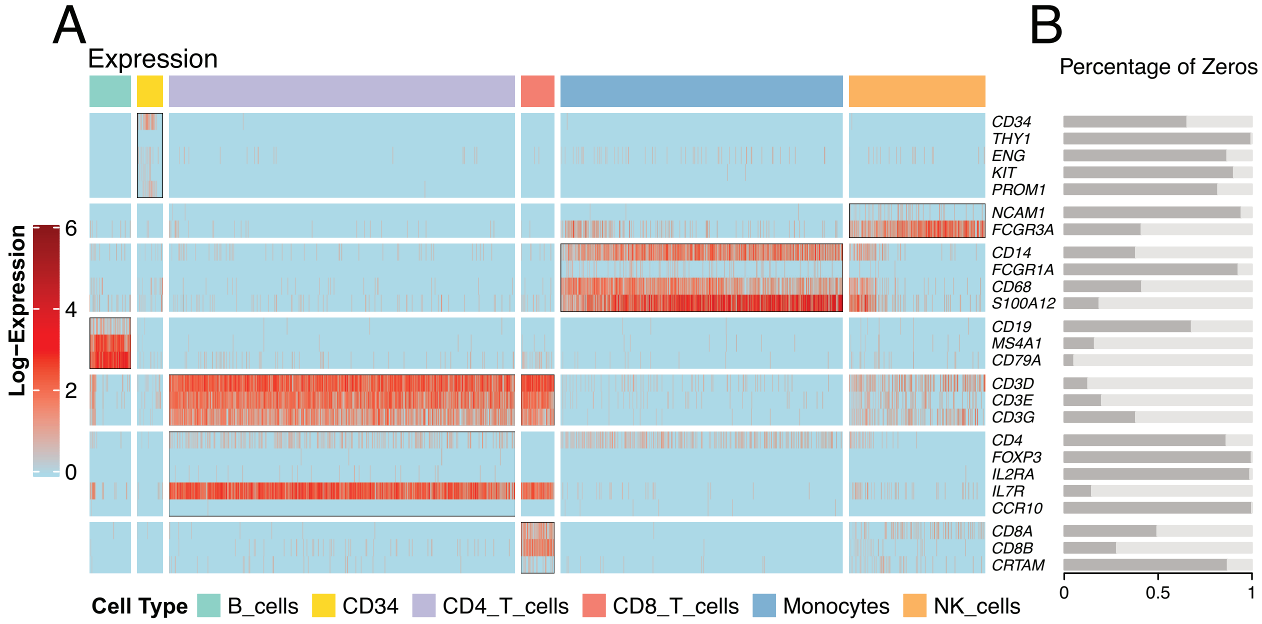

Figure S7: Visualization of CBMC data. A) Expression of cells from different cell types on marker genes. Columns are cells and rows are marker genes. Cells are grouped according to the (true) cell types that they belong to, with the colorful bar on the top of the expression matrix giving the cell type identities, and the marker genes are grouped according to the cell types that they correspond to. The white vertical and horizontal gaps separate cells from different types and marker genes corresponding to different cell types. The black boxes indicate the correspondences of cell types and marker genes. B) The bars show the percentages of cells that have zero expression in the corresponding cell type, for each marker gene.

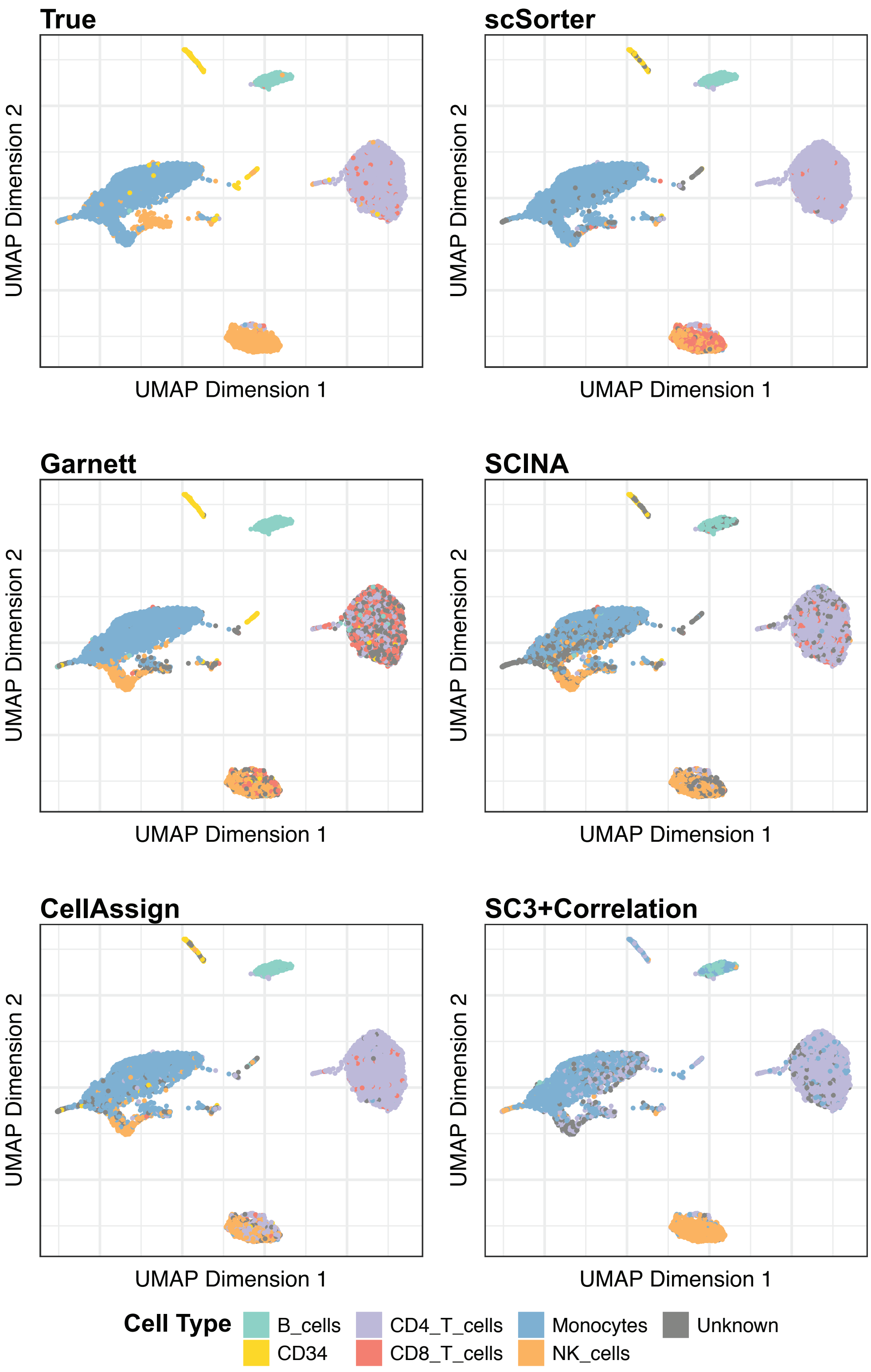

Figure S8: UMAP plots of CBMC dataset. The first panel is colored by true cell types. The rest five panels are colored by cell types assigned by the corresponding methods.

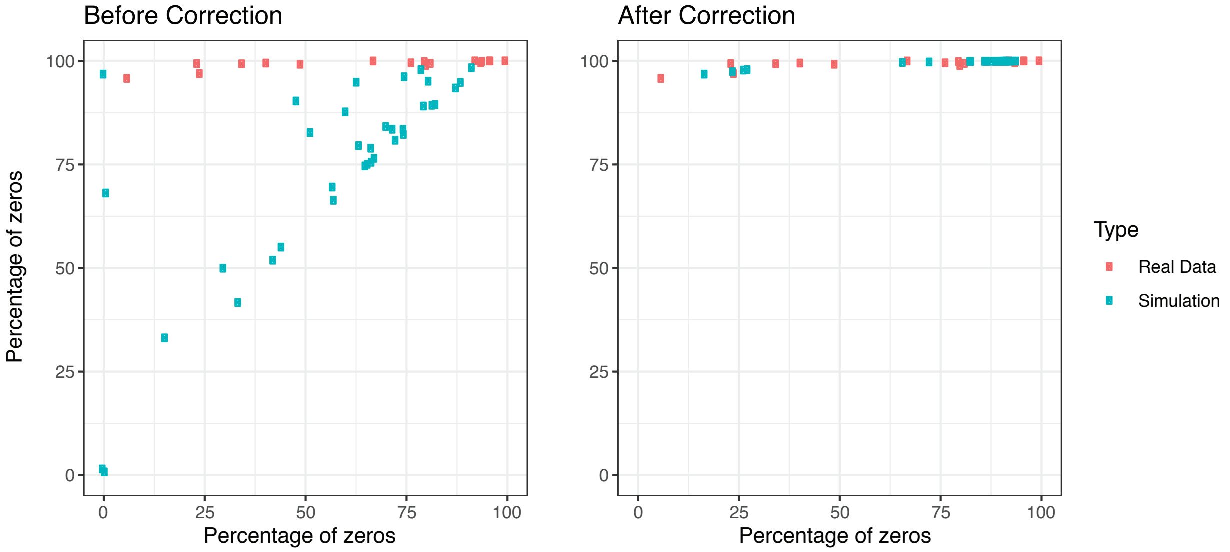

Figure S9: The percentage of zeros observed on marker genes in the cell type where these genes are marker genes (x axis) and the percentage of zeros observed on marker genes in the cell types where these genes are not marker genes (y axis), in PBMC data (left) and a simulated data (right).

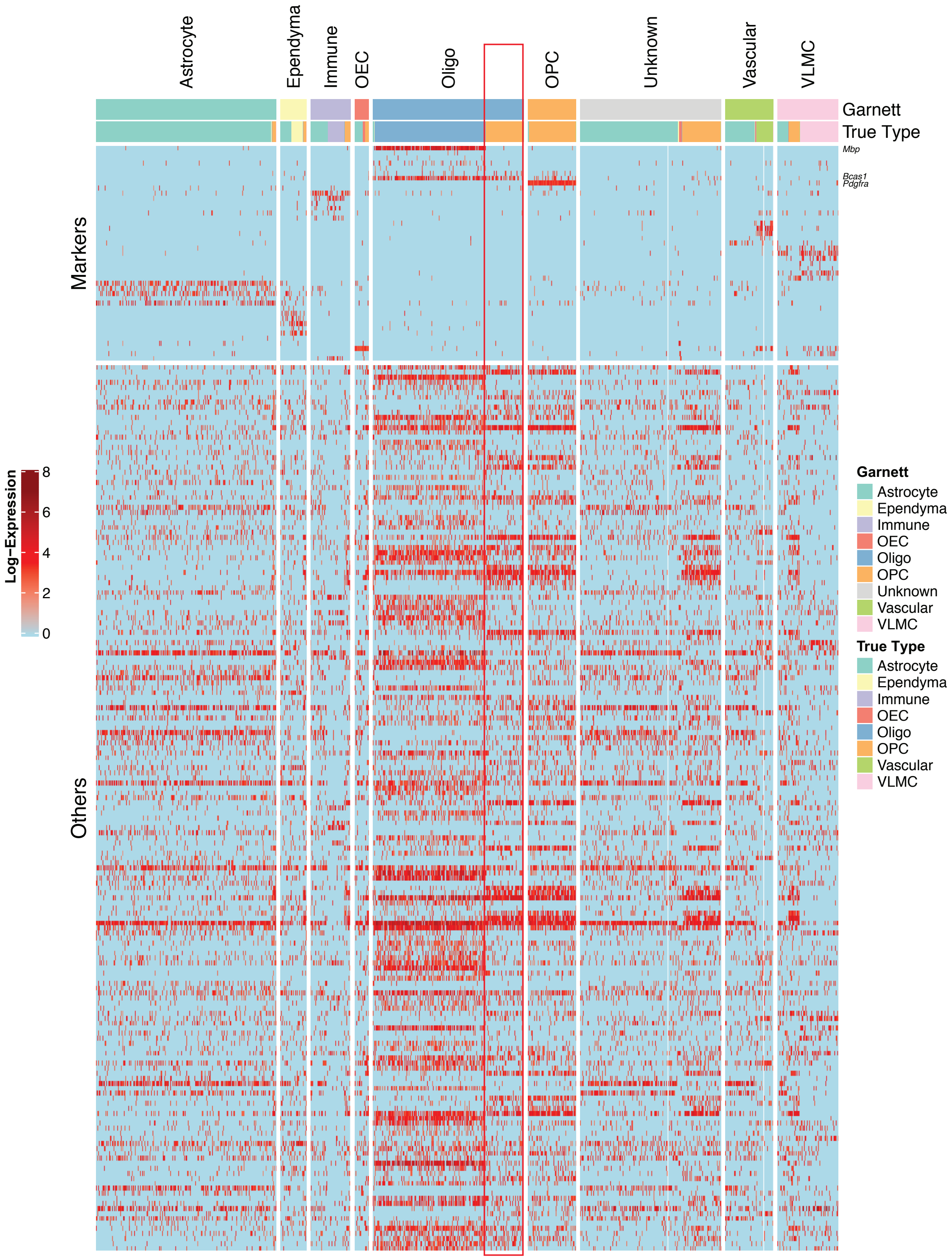

Figure S10: Heatmap of Rosenberg dataset labeled with true cell type and cell type assigned by Garnett. The red rectangle contains OPC cells that were assigned to Oligo by Garnett.

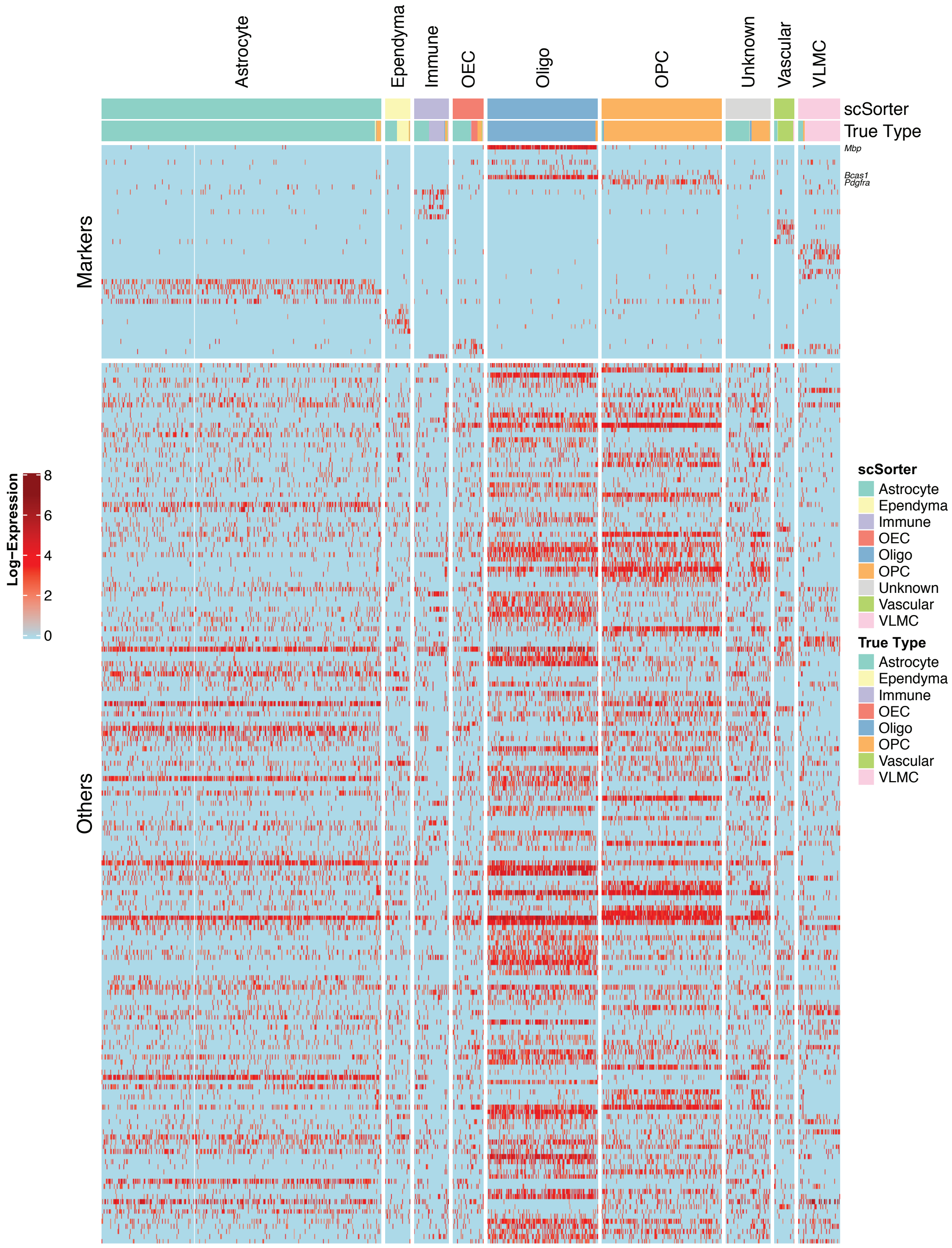

Figure S11: Heatmap of Rosenberg dataset labeled with true cell type and cell type assigned by scSorter.
