# Supplementary material for: scSorter: assigning cells to known cell types according to marker genes
Source: Genome Biol. 2021 Feb 22;22:69. doi: 10.1186/s13059-021-02281-7 (PMC7898451; doi:10.1186/s13059-021-02281-7)
Supplement: Supplementary file 2 — Additional file 2 Review history. [file 13059_2021_2281_MOESM2_ESM.docx]

Review History

**First round of review**

**Reviewer 1**

**Were you able to assess all statistics in the manuscript, including the appropriateness of statistical tests used?**

Yes: I was able to assess all statistics in the manuscript

**Were you able to directly test the methods?**

Yes

**Comments to author:**

Gui & Li present a semi-supervised approach to assign cell types to scRNA-seq data. Here "semi-supervised" means that the idendity of marker genes must be known, but no quantitative values for them. Their method is an extension of the K-means algorithm to specifically include the marker genes into the clustering, with a subsequent step to find outliers to remove from clusters and call them "unassigned cell type".

The problem addressed is important and timely, the software is freely available, user-friendly and well documented.

Major issues:

- scSorter does not take the hierarchy of cell types into account. E.g. the T cells in the PBMC data described in lines 10-18 on page 6 were categorized into CD4+ and CD8+ in the Garnett paper, but this was not done here (this is also not consistent with what is said in line 18). I wonder whether the flat clustering scheme in scSorter has problems with subtypes? This should be evaluated and compared to the other approaches.

- A simple, clustering-based baseline approach should be included: Perform Louvain clustering (different principal components and resolutions), assign most parsimonous cell type to clusters based on average marker gene expression (like in the CellAssign paper)

- A validation based on CITE-seq data should be included. All real data are based on manual annotation, i.e. clustering + manual marker gene assignment. Clustering-based approaches might appear superior to other methods just because of that.

- Simulated data were generated in a very ad-hoc manner from Normal distributions with dropouts drawn from some logistic curve. This is an oversimplification and favors scSorter (which essentially assumes a Normal distribution due to the sum-of-squares penalty). The other methods expect UMI data (i.e. counts). The authors should use Splatter and the extensions developed in the CellAssign paper.

- The second step in the 2-step procedure (detection of the "unassigned" type cells) seems to be flawed: If the "unassigned" type cluster is large, it will attract one of the cluster centroids. For that, (i) scSorter "underclusters" the known cell types with unpredictable results, and (ii) all cells from the unassigned type will be close to the cluster centroid and will not be filtered out. I would like to see an analysis with increasing sizes of the "unassigned" type cluster (preferably with real data, see next point).

- The "unassigned" type detection is only evaluated using the simulated data. For the real data sets, the markers for each cell type should be removed (one cell type after the other) to evaluate this also on real data.

- The updates of \delta and \mu are flawed: Due to the min, the function is non-differentiable. The solution only includes factors for the clusters where the minimum equals x-\mu-\delta for the current assignment [I did not check the other algorithms].

Minor issues:

- While generally well-written, the text needs some improvement (Structure: The introduction is overly long. E.g. the analysis of the dropouts of the marker genes could be moved to the results section. Language: "Last but not least", "novel thing" "users may have less head-scratching",...)

- The discussion of prior iterations of the clustering scheme is hard to understand without having gone through the methods part. Move?

- Theoretical point: All cluster centroids can be freely moved by the algorithm, except for the coordinates for the marker genes. For these there is a single "base level" (mainly defined by all other clusters), and an elevated level (defined by the "high expressers" in the cell type cluster). It would be nice to read some discussion on this decision (you could also have them moving freely for all other clusters, and the mean of all +\delta or the mean for the cell type cluster; my guess is that this wouldn't change the clustering a lot if at all, and maybe there are other reasons).

**Reviewer 2**

**Were you able to assess all statistics in the manuscript, including the appropriateness of statistical tests used?**

Yes: I have made a high level overview of the statistical methodology presented

**Were you able to directly test the methods?**

No

**Comments to author:**

In this paper, Guo and Li introduce scSorter, a computational method for the assignment of scRNA-seq data to known and unknown cell types. The work builds upon existing methods that attempt to map cells as measured by scRNA-seq to a priori known cell types. In contrast to reference based approaches, the current work focusses on marker (or semi supervised) based approaches, where it is known that certain cell types over express certain markers, but the exact over-expression is unknown.

The novelty in the presented work is the inclusion of non-marker genes in the classification problem. The logic is that certain markers may in fact be poor markers of a given cell type (i.e. lowly detected) and by leveraging additional genes the cell type assignment can be sharpened. The approach therefore borrows strength from additional genes by including them in the clustering.

The authors introduce their method and demonstrate it on a range of real and simulated datasets, showing general superiority to existing works. While the proposed approach is timely and interesting, I believe there are several ways in which the manuscript could be much improved, as detailed below.

\# Major

For the real data examples, how are the set of non-marker genes selected? Is it all genes detected in the experiment, or only those with high variance? Can the authors demonstrate the method is robust to this choice but e.g. varying the top genes (as per variance or otherwise) selected for the "non-marker" genes?

In the discussion of marker genes, the authors write "some marker genes are expected, biologically, to be expressed at low levels in some cells in the corresponding cell type." but this directly contradicts their definition of a marker gene: "marker genes for a cell type are genes that are over-expressed (i.e., have higher expression levels) in that cell type". Given this, can the authors rule out that their observation about the low expression of marker genes is simply because the genes are poor markers of the cell types of interest?

Simulations setup: in the simulated data, each cell type has 2-5 marker genes + 100 non marker genes. However, I'm not sure where this overall number (~105) of genes comes from, given the transcriptome is >20k genes and a regular scRNA-seq experiment will detect around 10k genes in total. Furthermore, the noise model of the simulations seems wrong: scRNA-seq data is count data, commonly modeled either with negative binomial or multinomial distributions. The authors use a gaussian distribution with means drawn from Unif(2,5), meaning (i) negative expression values are possible, (ii) adding excess zeros doesn't make sense to model dropout since the minimum possible expression value is not 0. Can the authors (i) re-do their simulations with a realistic number of genes (10k), (ii) use a realistic generative model for the simulations (e.g. splatter https://github.com/Oshlack/splatter).

Metrics: since this is essentially a classification problem, can the authors use a more standard metric of accuracy such as area under the receiver operator curve or area under the precision recall curve, rather than 1-accuracy, which

Ground truth in comparisons: the authors use previously specified cell types as ground truth, which isn't technically correct given these cell types are often inferred from the data typically via clustering algorithms which themselves have multiple choices with a subjective interpretation, i.e. the ground truth for comparisons is based on the approach the authors criticize in the introduction as problematic. This is especially an issue when comparing accuracies, e.g. the performance of different algorithms. The authors state that Garnett incorrectly assigns OPC cells to Oligo types and interpret this because "even when some marker genes are expressed (especially, Mbp and Bcas1 for Oligo, as shown in Figure 1),", in other words, if the cells assigned as Oligo by Garnett express Oligo markers, where is the justification that these are not truly Oligo? The fact that they cluster with the OPC cells in the original analysis does not establish cell identity, and an alternate clustering may change this.

Lack of comparison to CellAssign: the authors state that they were unable to achieve consistent results from CellAssign but also state that log normalized counts where used as input, when CellAssign expects raw counts. Can the authors check they are using CellAssign correctly? The fact they find it doesn't work at all is at odds with both its publication and subsequent usage.

Robustness to random initializations: presumably the optimization problem is non-convex. Can the authors demonstrate robustness to multiple initializations?

\# Minor

Human cell atlas is missing a citation (page 1 line 47)

Page 1 line 48: Seurat itself isn't a clustering algorithm but uses leiden clustering on a nearest neighbor graph

Data presentation: can the authors use boxplots or violin plots rather than bar charts with errors?

**Authors Response**

**Point-by-point responses to the reviewers’ comments:**

We thank the reviewers for the very insightful comments. In response, we have done additional analyses and edited the main text and Supplementary Materials accordingly (changes are highlighted).

Response to Referee 1

Guo & Li present a semi-supervised approach to assign cell types to scRNA-seq data. Here “semi-supervised” means that the identity of marker genes must be known, but no quantita¬tive values for them. Their method is an extension of the K-means algorithm to specifically include the marker genes into the clustering, with a subsequent step to find outliers to re¬move from clusters and call them “unassigned cell type”.

The problem addressed is important and timely, the software is freely available, user-friendly and well documented.

Major issues:

Q1- scSorter does not take the hierarchy of cell types into account. E.g. the T cells in the PBMC data described in lines 10-18 on page 6 were categorized into CD4+ and CD8+ in the Garnett paper, but this was not done here (this is also not consistent with what is said in line 18). I wonder whether the flat clustering scheme in scSorter has problems with subtypes? This should be evaluated and compared to the other approaches.

*A1 - We apologize for the inaccurate and inconsistent description. Indeed, while the Garnett paper uses two subtypes of T cells, CD4+ and CD8+, to demonstrate its ability in dealing with hierarchy of cell types, we did not specify the subtypes. We have changed our description in the “Description of real datasets” section to make this clear. The complete lists of marker genes for real datasets are available in Supplementary Materials.*

*To see how scSorter performs on data where hierarchy of cell types is present, we ran scSorter on PBMC data with CD4+ and CD8+ treated as two different cell types, with the marker genes for CD4+ being the marker genes specifically for CD4+ in addition to the marker genes for T cells, and the marker genes for CD8+ being the marker genes specifically for CD8+ in addition to the marker genes for T cells. Also, we still simply set all the weights for marker genes as w0 = 2.*

*The misclassification rate for scSorter, Garnett, SCINA, and CellAssign were 0.1302, 0.0819, 0.3157, and 0.6739, respectively. We see that Garnett indeed outperformed scSorter in this case, although the performance of scSorter was still acceptable (and much better than that of SCINA and CellAssign). Moreover, scrutinizing into the performance of scSorter and Garnett, scSorter misclassified 13.0% of CD4+ T cells and 11.7% of CD8+ T cells, while Garnett misclassified 1.7% of CD4+ T cells and 15.8% CD8+ T cells. We see that Garnett did not perform better in all cell types. We also would like to argue that while scSorter does not take advantage of cell type hierarchy, we also do not see particular “problems” of its algorithm that prevent it from working decently under cell type hierarchy.*

*We surely agree with the reviewer that this is a valuable problem to mention, and so we reported the performance of different methods on the cell type hierarchy case on PBMC data in the Discussion section of the main text.*

Q2- A simple, clustering-based baseline approach should be included: Perform Louvain clustering (different principal components and resolutions), assign most parsimonious cell type to clusters based on average marker gene expression (like in the CellAssign paper)

*A2 - We appreciate the reviewer’s idea. We implemented such a two-step, clustering-based method and compared it with scSorter on both simulated and real data. Again, scSorter showed much higher accuracy than this method. We described how we implemented this method and its performance in detail in Supplementary Materials (section “A straightforward two-step approach”) and discussed it in the Discussion section of the main text.*

Q3- A validation based on CITE-seq data should be included. All real data are based on manual annotation, i.e. clustering + manual marker gene assignment. Clustering-based approaches might appear superior to other methods just because of that.

*A3 - This is a great suggestion. We added a fifth real dataset—a cord blood mononuclear cells (CBMC) dataset generated by Stoeckius et al. [1] using CITE-seq on the 10x platform. The description of the data is given in the last paragraph of the “Description of real datasets” section of the main text, and the results are given in the “Results” section of the main text. As a recap, Table RL1 gives the misclassification rates of different methods, including the straightforward two-step approach suggested by the reviewer in Q2. We see that in this data, scSorter still gives the highest accuracy.*

*Method	scSorter Garnett	SCINA	CellAssign	Q2-suggested*

*Misclassification Rate 0.1551	0.3986	0.2391	0.1816	0.3014*

*Table RL1: Misclassification rate of different methods on CBMC data.*

Q4- Simulated data were generated in a very ad-hoc manner from Normal distributions with dropouts drawn from some logistic curve. This is an oversimplification and favors scSorter (which essentially assumes a Normal distribution due to the sum-of-squares penalty). The other methods expect UMI data (i.e. counts). The authors should use Splatter and the extensions developed in the CellAssign paper.

*A4 - This is a great suggestion. We have updated the simulation section of our manuscript. In this revised version, the simulated data were generated using Splatter with the extensions developed in the CellAssign paper and further corrections to better describe the excessive zeros observed in real data. The details are given in the “Details on simulated data” section of Supplementary Materials.*

*The performance of scSorter as well as other methods are described in the “Results” section of the main text. We see that in these new simulated data, scSorter still gave the lowest misclassification rate among all methods across all simulation scenarios/settings. On average, the misclassification rate of scSorter was 34.1% lower than that of Garnett, 33.1% lower than that of SCINA, 46.5% lower than that of CellAssign, and 41.3% lower than that of the approach suggested in Q2.*

Q5- The second step in the 2-step procedure (detection of the “unassigned” type cells) seems to be flawed: If the “unassigned” type cluster is large, it will attract one of the cluster centroids. For that, (i) scSorter “underclusters” the known cell types with unpredictable results, and (ii) all cells from the unassigned type will be close to the cluster centroid and will not be filtered out. I would like to see an analysis with increasing sizes of the “unassigned” type cluster (preferably with real data, see next point).

*A5 - The reviewer raised a valuable point. For a cell to be assigned to a particular cluster, which is supposed to correspond to a particular known cell type, it should have high ex¬pression on marker genes and/or its expression on other genes should be close to cells that have high expression on marker genes. And since we typically set w0 to be larger than 1 (In our paper, we set w0 = 2), the distance to a centroid will still be largely determined by cells that have high expression on marker genes, i.e., those “known” cells. Meanwhile, since min is used, the estimate of the centroid on the marker genes is unlikely to bias down seriously. Thus, the “unassigned” type of cells, even if they are of large number, is not likely to at¬tract a cluster centroid. But we agree that the reviewer’s concern is quite meaningful (and thoughtful), and so we followed the suggestion and conducted an analysis with large sizes of the “unassigned” type cluster on a real dataset. The results is provided in our answer to the next question.*

Q6- The “unassigned” type detection is only evaluated using the simulated data. For the real data sets, the markers for each cell type should be removed (one cell type after the other) to evaluate this also on real data.

*A6 - We used PBMC data to evaluate scSorter on the “unknown” type detection. This data is highly imbalanced, i.e., the numbers of cells in different cell types differ a lot, and thus it is ideal for this task. We chose three cell types that represented different cell type sizes (the percentages of cells in these cell types are shown in Table RL2; note that the T cells account for as many as 67.97% of all cells) and removed the marker gene information for one cell type at a time. We wanted to see whether scSorter could correctly call these cells as “unknown”. Table RL2 gives the overall misclassification rate. We see that comparing with using the original data (shown in the table as “None” cell type was removed), the misclassification rate of removing any of the three cell types was not significantly larger. This suggests that scSorter is capable in calling “unknown” cell types, even if there is a large proportion of “unknown” cells.*

*Cell type removed Percentage of cells Misclassification rate*

*None	0%	0.0608*

*Monocytes	2.76%	0.0528*

*B cells	10.65%	0.0637*

*T cells	67.97%	0.0677*

*Table RL2: Misclassification rate of scSorter on the PBMC dataset when a cell type is re¬moved.*

Q7- The updates of 8 and 1a are flawed: Due to the min, the function is non-differentiable. The solution only includes factors for the clusters where the minimum equals x-1a-8 for the current assignment [I did not check the other algorithms].

*A7 - The reviewer is right! And it is truly insightful to point this out. Indeed, due to the minimum, the function is non-differentiable. It is actually everywhere continuous but only piece-wise differentiable. Therefore, the formula for updating 8ik or 1ai we have derived by letting the first derivative equal zero is only a local stationary point instead of the global optimum. Actually, we have further shown (see updated Supplementary Materials) that the second derivative is positive, and hence the formula gives a local minimum point (at each updating step). This local minimum point may not be the global minimum point (at each updating step), which could also be one of the non-differentiable points.*

*We do not worry too much about the local instead of global minimum solution at each updating step because of two reasons. First, even if the global minimum point is identified and used at each updating step, due to the non-convexity of the optimization problem that scSorter tries to solve, the solution resulting from the whole iterative algorithm is still a local minimum, anyway. Second, the current formulas (8ˆik and ˆi) seem to be highly inter¬pretable: 8 is updated by the mean of the differences between the observed expression and the baseline expression in the cells with elevated expression, and is the mean expression level of all cells, with the elevated component of expression removed.*

*We have updated Supplementary Materials to make the derivation more clearly. We appreciate the reviewer for pointing out this problem so that we can correct our previous erroneous notations and assertions. The formulas we use for updating 8 and , though, do not need to be changed.*

Minor issues:

Q8- While generally well-written, the text needs some improvement (Structure: The introduction is overly long. E.g. the analysis of the dropouts of the marker genes could be moved to the results section. Language: “Last but not least”, “novel thing” “users may have less head-scratching”,...)

*A8 - Thank you for pointing these out. We have made the corresponding changes, including moving the analysis of the dropouts of the marker genes to the Results section. (This move is not highlighted in the main text as too many lines are involved.) Now we think the Introduction section and other sections are more balanced.*

Q9- The discussion of prior iterations of the clustering scheme is hard to understand without having gone through the methods part. Move?

*A9 - This is indeed the case! We have moved this discussion to the end of the Methods section of the main text. (This move is not highlighted in the main text as too many lines are involved.)*

Q10- Theoretical point: All cluster centroids can be freely moved by the algorithm, except for the coordinates for the marker genes. For these there is a single “base level” (mainly defined by all other clusters), and an elevated level (defined by the “high expressers” in the cell type cluster). It would be nice to read some discussion on this decision (you could also have them moving freely for all other clusters, and the mean of all +8 or the mean for the cell type cluster; my guess is that this wouldn’t change the clustering a lot if at all, and maybe there are other reasons).

*A10 - This comment is quite thoughtful. Indeed, there is a difference in “mobility” (flex¬ibility) of the coordinates for the marker genes and non-marker genes. We actually had extensive debates and trials on such approaches before we settled down with scSorter. We think the first and second alternative approaches that we have tried, which are presented in the end of the Methods section of the main text (previously in the Discussion section of the main text), may answer this question. Both alternatives work much worse than scSorter, and we have briefly discussed the reasons in that section.*

Response to Referee 2

In this paper, Guo and Li introduce scSorter, a computational method for the assignment of scRNA-seq data to known and unknown cell types. The work builds upon existing methods that attempt to map cells as measured by scRNA-seq to a priori known cell types. In contrast to reference based approaches, the current work focuses on marker (or semi supervised) based approaches, where it is known that certain cell types over express certain markers, but the exact over-expression is unknown.

The novelty in the presented work is the inclusion of non-marker genes in the classifica¬tion problem. The logic is that certain markers may in fact be poor markers of a given cell type (i.e. lowly detected) and by leveraging additional genes the cell type assignment can be sharpened. The approach therefore borrows strength from additional genes by including them in the clustering.

The authors introduce their method and demonstrate it on a range of real and simulated datasets, showing general superiority to existing works. While the proposed approach is timely and interesting, I believe there are several ways in which the manuscript could be much improved, as detailed below.

#Major

Q1 - For the real data examples, how are the set of non-marker genes selected? Is it all genes detected in the experiment, or only those with high variance? Can the authors demonstrate the method is robust to this choice but e.g. varying the top genes (as per variance or other¬wise) selected for the “non-marker” genes?

*A1 - We only used the highly variable genes. In the clustering/classification analysis of single-cell RNA-seq data analysis, filtering out low-variance genes is recommended as their signal-to-noise ratio is low and including them often hurts instead of benefits the analysis. The particular approach we used for our real data analysis was the “vst” method under Find- VariableFeature() function in the Seurat package, which is the default and recommended choice.*

*To see whether scSorter is robust to this choice, we tried two other methods in Seurat, “mean-variance-plot” and “dispersion”, and summarized the results in Table RL3. We see that the misclassification rate did not change much, suggesting that the performance of scSorter is rather stable with different choices of highly variable genes.*

*We have added the results to the Discussion section of the main text.*

*Dataset	Method*

*vst	mean-variance-plot	dispersion*

*Rosenberg	0.1746	0.1826	0.1646*

*TM pancreas	0.0550	0.0537	0.0537*

*MCA lung	0.0831	0.1000	0.0844*

*PBMC	0.0608	0.0597	0.0672*

*CBMC	0.1551	0.1629	0.1574*

*Table RL3: Misclassification rate of scSorter under different methods for choosing highly variable genes on real datasets.*

Q2 - In the discussion of marker genes, the authors write “some marker genes are expected, biologically, to be expressed at low levels in some cells in the corresponding cell type.” but this directly contradicts their definition of a marker gene: “marker genes for a cell type are genes that are over-expressed (i.e., have higher expression levels) in that cell type”. Given this, can the authors rule out that their observation about the low expression of marker genes is simply because the genes are poor markers of the cell types of interest?

*A2 – Good point! Our writing was confusing. We have edited our writing as follows: “First, some marker genes are expected, biologically, to be expressed at high levels only in a pro¬portion, but not all, of the cells in the corresponding cell type. These genes are expected, biologically, to be expressed consistently at low levels in other cell types, and thus they are still valid markers.” Together with the example of the maker genes of the Oligo cell type (“as the Oligo cells form a lineage, and these marker genes are known to express only at certain intermediate stages of oligodendrocyte development.”), hopefully now the meaning is clear.*

Q3 - Simulations setup: in the simulated data, each cell type has 2-5 marker genes + 100 non marker genes. However, I’m not sure where this overall number ( 105) of genes comes from, given the transcriptome is >20k genes and a regular scRNA-seq experiment will de¬tect around 10k genes in total. Furthermore, the noise model of the simulations seems wrong: scRNA-seq data is count data, commonly modeled either with negative binomial or multinomial distributions. The authors use a gaussian distribution with means drawn from Unif(2,5), meaning (i) negative expression values are possible, (ii) adding excess zeros doesn’t make sense to model dropout since the minimum possible expression value is not 0. Can the authors (i) re-do their simulations with a realistic number of genes (10k), (ii) use a realistic generative model for the simulations (e.g. splatter https://github.com/Oshlack/splatter).

*A3 – This is a great suggestion, and Reviewer 1 also raised a similar request (Q4). We have re-done our simulation accordingly and also updated our manuscript. Please also refer to our answer (A4) to Reviewer 1. Briefly speaking, under the new simulation framework, scSorter still significantly outperformed all other methods across all simulation scenarios/settings.*

Q4 - Metrics: since this is essentially a classification problem, can the authors use a more standard metric of accuracy such as area under the receiver operator curve or area under the precision recall curve, rather than 1-accuracy.

*A4 - We appreciate the reviewer’s suggestion, although the measures suggested are typi¬cally used for two-class classification problems. For multi-class problems, which are what we consider in this paper (there are typically more than two types of cells in a dataset), misclassification rate is the standard and most commonly used way for measuring perfor¬mance of classifiers [2]. Moreover, misclassification rate = 1- accuracy, and accuracy has been widely used to evaluate the performance of cell type assignment by previous studies [3, 4, 5, 6]).*

Q5 - Ground truth in comparisons: the authors use previously specified cell types as ground truth, which isn’t technically correct given these cell types are often inferred from the data typically via clustering algorithms which themselves have multiple choices with a subjective interpretation, i.e. the ground truth for comparisons is based on the approach the authors criticize in the introduction as problematic. This is especially an issue when comparing accuracies, e.g. the performance of different algorithms. The authors state that Garnett incorrectly assigns OPC cells to Oligo types and interpret this because “even when some marker genes are expressed (especially, Mbp and Bcas1 for Oligo, as shown in Figure 1),”, in other words, if the cells assigned as Oligo by Garnett express Oligo markers, where is the justification that these are not truly Oligo? The fact that they cluster with the OPC cells in the original analysis does not establish cell identity, and an alternate clustering may change this.

*A5 - We thanks the reviewer for raising this issue up. First and foremost, we completely* *agree with the reviewer on the point that the ground truth in comparisons is questionable and this may limit the validity/effectiveness of comparisons on real datsets. This is without doubt. But this is a common problem/difficulty for all methodological papers of such type and is hard to get around. We did use simulational data, where the true answers are known, although we have to admit that there could still be biases in comparisons using simulated data, as the conclusions could depend on how the data were simulated. Both reviewers pointed out potential problems in our previous simulational study and suggested better ways, which we followed in this revised submission. Hopefully the results on the newly simulated data could mitigate the reviewer’s concern.*

*Regarding the specific example of the OPC and Oligo cell types in Rosenberg data, we present a more detailed analysis here. Figure RL1 shows the heatmap of marker genes (of any of the known cell types) as well as highly variable genes, with the cells grouped according to the labels given by Garnett. Also given in the figure are the “true” cell types, the cell types that were determined by the original paper that generated the data. As the reviewer pointed out, these “true” cell types do not have to be the biological truth. The red rectangle highlights the “misclassified” OPC cells by Garnett, i.e., the cells that have “true” cell type OPC but were labeled as Oligo by Garnett.*

*In the figure, the top nine marker genes are for the Oligo or the OPC cell types. We marked out Mbp (a marker gene for the Oligo cell type), Bcas1 (a marker gene for the Oligo cell type), and Pdgfra (a marker gene for the OPC cell type), which are the only ones that have high expression in a significant proportions of cells. We see that overall the pre-assumed marker genes do not contain strong and consistent signals in differentiating the Oligo and OPC cell types. This is even more clearly the case for the cells in the rectangle: their expression levels on Mbp and Pdgfra are both consistently low, and only about one half of them have high expression on Bcas1.*

*However, the expression on the highly variable genes may give a more definite answer to the cell type assignment of the cells in the red rectangle. The expression profiles of these cells on the highly variable genes are highly similar to the OPC cells and very different from the Oligo cells. The contraction is so big, in our eyes, that the assignment of these cells to the OPC cell type is almost indisputable. That means that the “true” cell types of the Oligo and OPC cells are likely to be correct. Figure RL2 shows the heatmap with the cells grouped according to the labels given by scSorter. This figure supports the observation and argument we just presented.*

*Of course, we agree with the reviewer that the biologically true cell types may never be known for sure merely based on computational approaches. Moreover, the biologically true cell types also depend on how one defines cell types. One may define the cell types merely based on a small set of marker genes. Alternatively, one may define the cell types based on the overall expression profiles of all/many genes; while emphasizing more on a small set of marker genes, other genes also contribute as genes in a genome are typically highly related with each other (They regulate one another and may form a complicated “network”). We lean towards the latter way of defining cell types, and thus we decided to use both marker genes and other genes for cell type assignment.*

*We thank the reviewer for pointing this out. Our previous description in the manuscript was surely not clear enough. We expanded it and also included the two figures in Supple¬mentary Materials.*

Q6 - Lack of comparison to CellAssign: the authors state that they were unable to achieve consistent results from CellAssign but also state that log normalized counts where used as input, when CellAssign expects raw counts. Can the authors check they are using CellAssign correctly? The fact they find it doesn’t work at all is at odds with both its publication and subsequent usage.

*A6 - We were actually quite frustrated when we could not get consistent results from Cel-lAssign. Thanks to the reviewer for urging us to re-check into CellAssign: by looking into the source code of CellAssign line by line, we finally figured out the reason! And yes, we did not use CellAssign correctly. CellAssign accepts a marker-gene list and an expression matrix as input. Internally, CellAssign transforms the list of marker genes into a design ma¬trix for further computation. During this transformation, quite unexpectedly, the order of the input genes is altered. This alternation is invisible to the end user, but it breaks the cor¬respondence between the marker genes in the list and the genes in the expression matrix. CellAssign does not detect this wrong correspondence, but simply output incorrect results. After fixing this bug, we are now able to provide the correct results from CellAssign. We added them to the “Results” section of the main text, and we also updated figures in the main text and Supplementary Materials to reflect the change. (It is worth noting that Cel-lAssign also accepts another format of inputs: an expression matrix and a design matrix. That is, in this format the design matrix is directly given by the user instead of transformed from the marker gene list. If we had used this format at the beginning, we would have got the correct results at the first time.)*

*Here we give a brief summary of the performance of CellAssign. On simulated data, CellAssign performed significantly worse than scSorter, with 61.8% to 97.5% higher mis¬classification rate than scSorter. On real datasets (under the new simulation framework suggested by both reviewers), the performance of CellAssign varied a lot when compared to scSorter. On TM pancreas data, MCA lung data, and CBMC data, it gave comparable re¬sults as scSorter (misclassification rate CellAssign vs. scSorter = 0.0512 vs. 0.0550, 0.0948 vs. 0.0831, and 0.1816 vs. 0.1551, respectively). However, on PBMC data and Rosenberg data, its performance was much worse than scSorter (misclassification rate CellAssign vs. scSorter = 0.7820 vs. 0.0608, and 0.6799 vs. 0.1757, respectively).*

*We were curious why CellAssign failed on PBMC data and Rosenberg data, and we be¬lieve we have figured out the reason: CellAssign seems to have difficulty in dealing with imbalanced datasets, that is, datasets that contain cell types of very different sizes (i.e., very different numbers of cells in different cell types). We illustrate this using PBMC data, which contains a cell type, T cells, that account for 67.97% of all cells. We subsampled these 64,341 T cells to smaller numbers so that the data was more balanced, and re-ran CellAs-sign. Table RL4 gives the misclassification rates under subsamplings with different numbers of T cells. It is clear that the performance of CellAssign was acceptable when the number of T cells was comparable with other cell types, but as the data got more imbalanced, the performance of CellAssign deteriorated quickly.*

*Number of T cells	9,686	19,686	29,686	39,686	49,686	59,686*

*Misclassification rate	0.2655	0.2220	0.2239	0.7570	0.7888	0.8129*

*Table RL4: Misclassification rate of CellAssign on PBMC data with different levels of imbal¬ance.*

Q7 - Robustness to random initializations: presumably the optimization problem is non-convex. Can the authors demonstrate robustness to multiple initializations?

*A7 - The reviewer is right: the optimization problem is non-convex, and thus the solution can depend on the initialization value. scSorter uses the same solution as what people use for K-means clustering: using multiple random initializations and then choosing the best solution. This should greatly reduce the randomness in the final result, although it still cannot guarantee that the global optimum is achieved every time we run scSorter. To study exactly how much randomness in the results of scSorter, we ran scSorter ten times using different random seeds, and the results are summarized in Table RL5. We see that scSorter is generally quite stable under different random seeds.*

*We have added this result to Supplementary Materials (Table S2).*

#Minor

Q8 - Human cell atlas is missing a citation (page 1 line 47)

*A8 - Thanks! Added.*

*Dataset	Mean Standard Deviation*

*Rosenberg 0.1928	0.0149*

*TM pancreas 0.0550	0.0000*

*MCA lung 0.0831	0.0002*

*PBMC	0.0608	0.0000*

*CBMC	0.1551	0.0000*

*Table RL5: Mean and standard deviation of the misclassification rate of scSorter on real datasets under ten different random seeds.*

Q9 - Page 1 line 48: Seurat itself isn’t a clustering algorithm but uses leiden clustering on a nearest neighbor graph

*A9 - Thank you! Corrected.*

Q10 - Data presentation: can the authors use boxplots or violin plots rather than bar charts

with errors?

*A10 - Thanks for the suggestion. We now use boxplots.*

References

[1]	Marlon Stoeckius, Christoph Hafemeister, William Stephenson, Brian Houck-Loomis, Pratip K Chattopadhyay, Harold Swerdlow, Rahul Satija, and Peter Smibert. Simultane¬ous epitope and transcriptome measurement in single cells. Nature methods, 14(9):865, 2017.

[2]	Trevor Hastie, Robert Tibshirani, and Jerome Friedman. The elements of statistical learn¬ing: data mining, inference, and prediction. Springer Science & Business Media, 2009.

[3]	Ze Zhang, Danni Luo, Xue Zhong, Jin Huk Choi, Yuanqing Ma, Stacy Wang, Elena Mahrt, Wei Guo, Eric W Stawiski, Zora Modrusan, et al. Scina: A semi-supervised subtyping algorithm of single cells and bulk samples. Genes, 10(7):531, 2019.

[4]	Hannah A Pliner, Jay Shendure, and Cole Trapnell. Supervised classification enables rapid annotation of cell atlases. Nature methods, 16(10):983–986, 2019.

[5]	Allen W Zhang, Ciara O’Flanagan, Elizabeth A Chavez, Jamie LP Lim, Nicholas Ceglia, Andrew McPherson, Matt Wiens, Pascale Walters, Tim Chan, Brittany Hewitson, et al. Probabilistic cell-type assignment of single-cell rna-seq for tumor microenvironment profiling. Nature methods, 16(10):1007–1015, 2019.

[6] Isabella N Grabski and Rafael A Irizarry. Probabilistic gene expression signatures iden¬tify cell-types from single cell rna-seq data. bioRxiv, 2020.

**Second round of review**

**Reviewer 1**

The authors comprehensively addressed most of my concerns. However, there are two remaining issues that need to be resolved before publication:

1. A fair comparison to a baseline method, and exceeding its "performance" w.r.t. a reasonable measure is the minimal requirement to publish a new computational tool. A suitable baseline method would be a procedure that can easily be implemented with existing tools. The readers (me included) really want to know, whether it is worth to invest the time for getting another software package to run. The authors now implement "A straightforward two-step approach", but both the way how this is done and how it is presented need improvement:

- The baseline approach they implemented is based on k-means clustering and Tracy-Widom statistics to automatically set the number of clusters k, and then assigning clusters to cell types based on marker gene expression with a stringent correlation threshold. Tracy-Widom might fail miserably for non-normal data, and the stringent clustering might produce many "unassigned" clusters (how many are not shown, so I cannot judge this). I explicitly proposed something else, for a good reason: In the literature, the common procedure is to follow the Seurat tutorial. This includes Louvain-clustering (which is considered superior to k-means), and trying different "resolutions" (leading to different numbers of clusters).

- This approach is briefly indicated in the discussion, and results are presented in the Supplementary Material (only the overall accuracies). This additional approach must be presented in the main text alongside with the four other methods (i.e. Figure 2+3 and corresponding supplementary figures).

2. The authors now use a more standard approach to simulate test data. However, they adapted it to introduce additional zero gene expression values for marker genes (a major promise of their approach is to be able to cope with that). This very likely is a critical parameter for the performance of any cell type identification approach, so special care must be taken not to bias the results. Figure S9 shows the percentages of zeros in real and simulated data for the utilized pbmc marker genes. Why are there more cyan than red points (i.e. marker genes in the simulated data vs. real data)? In addition, after correction, there appears to be an excess of marker genes with >90% zeros in the simulated data compared to the real data. Thus, the correction seems inappropriate (why fit logistic curves, just set them to zero with a fixed probability such that the expected number of zeros for a particular marker gene is equal to the observed number in real data), and the results of the simulation might be biased in favor of the new approach.

**Reviewer 2**

Overall the manuscript is much improved and the authors have addressed the majority of my concerns. However, I still have some queries, mainly related to language:

The authors now write "Some marker genes are expected, biologically, to be expressed at high levels only in a portion, but not all, of the cells in the corresponding cell type". However, I still don't see how this doesn't directly contradict the definition of a marker gene, which the authors have even previous defined as a gene that is overexpressed in a particular cell type. If a gene is not highly expressed in all cells of a given cell type, then surely it is not a marker of that cell type?

The descriptions of the novelty of scSort compared to competing methods still seems overstated. For example, it is stated that it is "novel" that scSorter uses non marker genes, when Garnett does in fact use them as is subsequently acknowledged. Similarly, when the authors describe this they say "As a result, if the first step is not efficient because of not using non-marker genes, the second step will be compromised anyway." but this is an unscientific statement - the "efficiency" of a given step of an algorithm is not defined nor compared against, and nothing shows that the second step of Garnett is "compromised", a concept which is similarly undefined. Again, there is a claim of novelty that scSorter is the first method "to explicitly allow marker genes to express at a low level in their corresponding cell types." but this is also not true - CellAssign can model markers has having no over expression compared to baseline levels. I would encourage the authors to ensure any claims of e.g. "efficiency" are empirically justified, and that text about competing methods is detailed correctly.

T cell markers: the authors use the different genes coding for CD3, can they confirm whether CD45 (PTPRC) is also used?

**Authors Response**

**Point-by-point responses to the reviewers’ comments:**

Response to Reviewer 1

Q1- A fair comparison to a baseline method, and exceeding its "performance" w.r.t. a reasonable measure is the minimal requirement to publish a new computational tool. A suitable baseline method would be a procedure that can easily be implemented with existing tools. The readers (me included) really want to know, whether it is worth to invest the time for getting another software package to run. The authors now implement "A straightforward two-step approach", but both the way how this is done and how it is presented need improvement: The baseline approach they implemented is based on k-means clustering and Tracy-Widom statistics to automatically set the number of clusters k, and then assigning clusters to cell types based on marker gene expression with a stringent correlation thresh-old. Tracy-Widom might fail miserably for non-normal data, and the stringent clustering might produce many "unassigned" clusters (how many are not shown, so I cannot judge this). I explicitly proposed something else, for a good reason: In the literature, the common procedure is to follow the Seurat tutorial. This includes Louvain-clustering (which is considered superior to k-means), and trying different "resolutions" (leading to different numbers of clusters). This approach is briefly indicated in the discussion, and results are presented in the Supplementary Material (only the overall accuracies). This additional approach must be presented in the main text alongside with the four other methods (i.e. Figure 2+3 and corresponding supplementary figures).

*A1 - Following the requirement by the reviewer, we have presented the baseline approach in the main text alongside with the four other methods.*

*However, for the baseline method, we believe using SC3 for the first step is a better choice over using Louvain clustering. (We have added the reason described below to Supplemen¬tary Materials of our paper, and also mentioned it briefly in the main text.) The reason is, in the majority of real datasets used in this study, the cell types and corresponding marker genes of these datasets are derived from clusters detected by Louvain algorithm, and there¬fore using Louvain algorithm for the first step may suffer from data-snooping bias and thus does not serve as a good “baseline”.*

*Here are some examples about how real datasets use Louvain clustering. Rosenberg data was processed with a Jaccard-Louvain algorithm [1], which computes k-nearest-neighbors for each cell based on principal components, then builds cell-cell similarity matrix with Jac-card index based on the number of shared neighbors between every cell, and finally conducts clustering using the Louvain algorithm. TM pancreas, MCA lung, and CBMC data were all analyzed following the recommended approach of Seurat, which also computes k-nearest-neighbor graph of each cell based on reduced principal components or projections from other dimension reduction methods, constructs a shared nearest neighbor graph by calcu¬lating the neighborhood overlap with Jaccard index and conducts clustering with Louvain algorithm as last.*

*We admitted in our previous response letter that it is without doubt that the less of ground truth may limit the validity/effectiveness of comparisons on real datasets. However, we also explained that this is a common difficulty for all methodological papers of such type and made our best efforts to make the comparison as fair as possible.*

*For the baseline method, we thus chose SC3, which is a powerful and highly popular method for clustering single-cell RNA-seq data, and it is different enough from Louvain clustering.*

*We would also like to respectfully mention that while we did not compare with a baseline method exactly as the reviewer suggested, we did compare with the three state-of-the-art methods, which are actually also the only published methods developed for the problem of consideration.*

Q2- The authors now use a more standard approach to simulate test data. However, they adapted it to introduce additional zero gene expression values for marker genes (a major promise of their approach is to be able to cope with that). This very likely is a critical parameter for the performance of any cell type identification approach, so special care must be taken not to bias the results. Figure S9 shows the percentages of zeros in real and simulated data for the utilized pbmc marker genes. Why are there more cyan than red points (i.e. marker genes in the simulated data vs. real data)? In addition, after correction, there appears to be an excess of marker genes with >90% zeros in the simulated data compared to the real data. Thus, the correction seems inappropriate (why fit logistic curves, just set them to zero with a fixed probability such that the expected number of zeros for a particular marker gene is equal to the observed number in real data), and the results of the simulation might be biased in favor of the new approach.

*A2 - The reviewer raises three questions/concerns: (1) Why are there more cyan than red points in Figure S9? (2) After correction, there appears to be an excess of marker genes with >90% zeros in the simulated data, and thus the correction seems inappropriate. (3) Why fit logistic curves?*

*The answer to the first question is that this is because the number of marker genes in the simulated data (cyan) and that in the real data (red) are not the same. In our simulation, two to five genes were selected as marker genes for each cell type, and thus the total number of marker genes was a random number. We have added this comment to our description in Supplementary Materials.*

*Given that there is no one-to-one relationship between the cyan points and the red points, the approach suggested by the reviewer, i.e., setting counts to zeros with a fixed probability such that the expected number of zeros for a particular marker genes is equal to the observed number in real data, will not work, and using logistic regression becomes a natural choice. This answers the third question from the reviewer.*

*For the second question, there are always randomness in the results, and every simulated data set is different. Also, in general there is no perfect way to generate simulated data; it may not be quite practical to require simulated data to perfectly mimic real data.*

Response to Reviewer 2

Q1 - The authors now write "Some marker genes are expected, biologically, to be expressed at high levels only in a portion, but not all, of the cells in the corresponding cell type". However, I still don’t see how this doesn’t directly contradict the definition of a marker gene, which the authors have even previous defined as a gene that is overexpressed in a particular cell type. If a gene is not highly expressed in all cells of a given cell type, then surely it is not a marker of that cell type?

*A1 - Our understanding is that, if a gene is not highly expressed in all cells in a given cell type, it may still be a marker gene of that cell type.*

*We actually give an explanation in our main text: “These genes are expected, biologically, to be expressed consistently at low levels in other cell types, and thus they are still valid markers.” That means, since these genes can still be used to differentiate cells that belong to this cell type and not belong to this cell type, they are still valid markers. In the main text, we also give an example of such marker genes: “An example is the marker genes for the Oligo cell type, which we just discussed.” The discussion we have in the previous paragraph is: “This is biologically meaningful, as the Oligo cells form a lineage, and these marker genes are known to express only at certain intermediate stages of oligodendrocyte development. For each marker gene, Figure 1B shows the percentage of cells where its expression is zero in its corresponding cell type. On average, this percentage is 72%.”*

*Actually, “marker genes” is a concept that is so widely used that different people may have different definitions/understandings about it and some of these definitions/under-standings may not be 100% biologically correct. For example, in many publications, marker genes are identified as genes that have distinct mean expression in a cell type. We could add these discussions to the place when the concept of “marker genes” is first introduced (fourth paragraph of Background), but this will make the paragraph read awkward.*

Q2 - The descriptions of the novelty of scSort compared to competing methods still seems overstated. For example, it is stated that it is "novel" that scSorter uses non marker genes, when Garnett does in fact use them as is subsequently acknowledged. Similarly, when the authors describe this they say "As a result, if the first step is not efficient because of not using non-marker genes, the second step will be compromised anyway." but this is an unscientific statement - the "efficiency" of a given step of an algorithm is not defined nor compared against, and nothing shows that the second step of Garnett is "compromised", a concept which is similarly undefined. Again, there is a claim of novelty that scSorter is the first method "to explicitly allow marker genes to express at a low level in their corresponding cell types." but this is also not true - CellAssign can model markers has having no over ex¬pression compared to baseline levels. I would encourage the authors to ensure any claims of e.g. "efficiency" are empirically justified, and that text about competing methods is detailed correctly.

*A2 – We thank the reviewer for the comments. We did the following to address them: (1) We changed “novel ideas” to “strategies,” and “novel idea” to “strategy.” (2) We removed the sentence of “As a result, if the first step is not efficient because of not using non-marker genes, the second step will be compromised anyway.” (3) To make the difference between scSorter and CellAssign in the second novelty/strategy more clear, we modified “at a low level in their corresponding cell types” to “at a low level in some cells from their corresponding cell types.”*

Q3 - T cell markers: the authors use the different genes coding for CD3, can they confirm whether CD45 (PTPRC) is also used?

*A3 – CD45 (PTPRC) is not used. As mentioned in the manuscript, the marker-gene list we used for T cells of PBMC and CBMC dataset was extracted without modification from the Garnett paper, which does not include CD45 (PTPRC).*

*References*

*[1] Karthik Shekhar, Sylvain W Lapan, Irene E Whitney, Nicholas M Tran, Evan Z Macosko, Monika Kowalczyk, Xian Adiconis, Joshua Z Levin, James Nemesh, Melissa Goldman, et al. Comprehensive classification of retinal bipolar neurons by single-cell transcrip-tomics. Cell, 166(5):1308–1323, 2016.*
